# Trade Wars and Election Interference

**DOI:** 10.1007/s11558-022-09464-2

**Published:** 2022-06-11

**Authors:** Ryan Brutger, Stephen Chaudoin, Max Kagan

**Affiliations:** 1 Travers Department of Political Science, University of California, Berkeley, USA; 2 Department of Government, Harvard University, Cambridge, USA

**Keywords:** Trade war, Tariffs, Retaliation, Election interference, Intervention, public opinion, F13, F14, F15, D72

## Abstract

In response to the Trump trade war, China, the EU, and other countries enacted politically-targeted trade retaliation (PTTR) against swing states and Republican strongholds in the United States. We argue that PTTR increases public concerns about foreign election interference and assess the effects of such retaliation across partisan affiliations. We test our predictions using a national survey experiment in the United States fielded before the 2020 election. In contrast to findings about sanctions and foreign endorsements, we find strong evidence that PTTR increases fears of election interference among *both* Republicans and Democrats. Partisan double standards in reaction to PTTR were strongest for retaliation targeting swing states and smaller for retaliation targeting the President’s base. Overall, the evidence shows that economic policies which are not primarily intended to influence elections may nevertheless come to be viewed by the public as foreign election interference.

**Supplementary Information:**

The online version contains supplementary material available at 10.1007/s11558-022-09464-2.

## Introduction

The trade war initiated by Donald Trump was one of the largest shocks to the international system since the Great Recession. The trade war began in early 2018 with the United States imposing tariffs on washing machines and dishwashers from Asian countries, and quickly escalated and expanded to myriad products and countries as the United States and its trading partners exchanged repeated rounds of retaliatory tariffs.

Apart from its magnitude, a central feature of the trade war was how tariffed goods were selected for *political* reasons—a phenomenon we refer to as politically-targeted trade retaliation (PTTR). While all economic statecraft is political to a certain degree, the economic pain of these sanctions appeared to be targeted in such a way as to maximize the resulting political pain, especially through partisan electoral channels. PTTR against the United States targeted products produced in specific locations with the intent to maximize political consequences for President Trump and his Republican allies. Some salvoes in the trade war targeted Trump’s base of support in conservative states. For instance, China’s retaliation focused heavily on agricultural products, such as soybeans, that are produced in pro-Trump strongholds.^1^ Other shots in the trade war targeted contested swing states that were viewed as pivotal for Republicans in the 2018 and 2020 elections. Foreign tariffs were particularly painful for exports produced in battleground states like Michigan and Wisconsin.^2^ Some retaliatory tariffs also sought to inflict political pain through non-electoral channels by applying leverage on key policymakers. For example, Chinese tariffs targeted products manufactured in the home districts of Speaker of the House Paul Ryan and Senate Majority Leader Mitch McConnell.^3^

While the Trump trade war provides an important recent example, PTTR is not a new phenomenon. For decades, the United States has strategically placed tariffs on specialty food products like cheese, wine, and sparkling water to gain leverage in trade negotiations with Europe.^4^ American trade negotiators specifically choose these products because their industries are politically influential in Europe, maximizing political leverage, but also because they did not constitute a major part of the average American consumers’ purchasing basket.^5^ China has also used trade policy strategically, placing tariffs on the powerful Australian coal and wine industries following disputes over imports of 5G technology and a proposed government inquiry into the origins of the COVID-19 virus.^6^ Although PTTR was not unique to the Trump trade war, the recent tariff retaliation represents an especially important case because of its large economic and political impact. Because of the magnitude of bilateral trade ties between the United States and China, China’s decision to use PTTR had significant political and economic effects on targeted US regions.^7^ The specter of future PTTR looms so large that the European Union has begun developing a suite of tools to counter economic coercion.^8^

Academic research on trade policy generally analyzes the electoral impact of trade policy through the lens of retrospective voting. Voters consider the impact of trade policy on either their personal financial situation or their sociotrope, then reward or punish the incumbent accordingly. In this paper, we look at a different way trade policy may affect domestic politics. Rather than focusing on whether trade policy affects the political fortunes of incumbents through economic channels, we consider whether PTTR may exacerbate public concerns about election interference and thus undermine trust in democratic institutions. Regardless of how it is intended by the countries which use PTTR, trade policy takes place in front of public audiences who are increasingly aware of foreign interference in democratic elections.^9^ Following public debates about Russian electoral interference in the 2016 Presidential election, worries about foreign election interference became an increasingly common feature of American political life. In a speech prior to the 2018 midterm election, Vice President Pence said that “China has initiated an unprecedented effort to influence American public opinion, the 2018 elections, and the environment leading into the 2020 presidential elections.”^10^ Notably, Pence did not suggest that China was directly interfering in elections through ballot-box tampering or electronic hacking, but that it was using its tariff policies for political gain and had “specifically targeted industries and states that would play an important role in the 2018 election.”^11^

Given the heightened salience of election interference in the United States, we argue that voters might draw the same conclusions as Vice President Pence in linking trade policy with election interference. The overtly political/electoral nature of tariff targets can trigger the type of worries associated with more direct attacks on electoral integrity or partisan foreign endorsements. Trade policy and the political machinations behind it may once have flown under the radar, but the increasing salience of foreign electoral interventions means that the distinction between the economics of trade policy and political interference can become blurred for some subsets of the population. While there is an expansive literature on economic retrospective voting and how it may affect incumbent candidates, we believe we are among the first to link the literatures on trade retaliation and election interference. Specifically, we argue that awareness of the politically-targeted nature of trade retaliation can affect public attitudes not just towards specific candidates or parties, but also concerns about the integrity of democratic elections more broadly.

In this paper, we first document how PTTR was not an esoteric or unknown feature of the trade war, pondered only by academics and elites. After the trade war began in 2018, the media provided extensive coverage highlighting the politically-targeted nature of foreign tariffs. In our survey of media coverage of the trade war, we find that over 30 percent of US newspaper stories covering the trade war specifically mention that swing states or Republican strongholds were targeted or disproportionately affected by retaliatory tariffs. This provides concrete evidence that PTTR has gained prominence in the media and political discourse.

Second, we develop our theory linking PTTR with worries about election interference. Although scholars have not generally analyzed PTTR as a form of foreign electoral interference, we show that PTTR fits within existing conceptions of election interventions and interference. As such, we lay out hypotheses that extend prior research on election interference to cover PTTR. We focus on two hypotheses: (1) that PTTR can increase concerns about election interference and (2) that reactions to PTTR will exhibit a partisan double standard, where the targeted group’s co-partisans react most negatively to PTTR.

We then use a survey experiment fielded to US respondents in the weeks before the 2020 presidential election to assess how different types of politically-targeted trade retaliation affect perceptions of electoral interference. To make the treatments realistic, we use similar language to that found in our survey of media reports. We also distinguish between two types of PTTR—base targeting and swing state targeting—which can trigger different reactions among subsets of the electorate. Using a survey experiment allows us to isolate the specific effect of political targeting apart from general exposure to knowledge about retaliatory tariffs. By comparing treatments that mention retaliation with and without additional information about political targeting, we can show that increased worries of electoral interference stem from the politically-targeted nature of PTTR, not simply from negative backlash towards tariffs in general. To make our research design more conservative, we use a vignette describing the trade war with the European Union, as opposed to China. Using a vignette about China might have made respondents even more likely to react negatively, given the larger degree of anti-China sentiment among Americans. Using the European Union, an organization not generally thought to be a US adversary, makes our research design a “harder case” for finding negative reactions to PTTR.

We find that PTTR has a strong effect on public concerns about foreign interference in the 2020 US presidential election. PTTR targeting either Trump’s base or electorally important swing states increases the portion of respondents concerned about election interference by 13 percentage points, compared to respondents who read about general, un-targeted retaliation.

Notably, *both* Democrats and Republicans shared this reaction, even though PTTR targeted Republicans (for retaliation aimed at base states) and President Trump’s political prospects (for retaliation aimed at swing states). This finding is in contrast to most existing research, which finds that respondents react more negatively to targeting of their co-partisans and care very little about foreign electoral interventions targeted at the opposing party.^12^ Instead of a partisan “double standard,” we find that both Republicans and Democrats have similar negative reactions to learning that trade retaliation targeted states Trump carried in the 2016 election. We do find small partisan differences in reactions to learning about retaliation targeting swing states. Republicans react more negatively to swing state targeting, but Democrats also exhibit negative reactions.

Our results demonstrate that even policies such as tariffs that do not directly interfere with the voting process may nonetheless be viewed by the public as a form of electoral interference. With this in mind, a full assessment of the costs of trade wars must go beyond an accounting of the quantifiable economic impact and the near-term political consequences for the politicians involved, but also encompass the long-term effect of deteriorating trust in democratic institutions. Given increasing economic nationalism on the one hand and heightened concerns about electoral integrity on the other, we suspect that neither PTTR nor fears of election interference will go away. As such, we feel that research examining the connections between trade policy and election interference is important for our field.

## Media coverage of politically targeted trade retaliation

Trump imposed tariffs on imports from China, the EU, and many other countries in early 2018. Retaliation was swift. Countries retaliated by targeting geographic areas that formed the base of President Trump’s 2016 electoral support. Virtually every country targeted by Trump’s Section 232 tariffs retaliated against agricultural goods produced in states and counties that Trump carried handily in the 2016 elections.^13^ For example, the EU retaliated with 25 percent tariffs against corn, rice, and peanuts, produced in states like Iowa, Arkansas, and Georgia respectively.^14^

Other prongs of retaliation against Trump’s tariffs targeted goods produced in electorally competitive areas, i.e. swing states. Countries which levied PTTR against swing states hoped that the economic consequences of the trade war would translate into political pain for the political leaders on the other side of the trade war. For a geostrategic opponent facing elections, such as Donald Trump in 2020 (or Republicans facing mid-term elections in 2018), political pain for voters in swing states can translate directly into electoral losses. Since many agricultural products are also produced in swing states, a number of these products were also in the crosshairs. A Deutsche Bank report emphasized that Chinese retaliation “has been on agricultural producers and agriculture products, which happen to be in swing states.”^15^ The BBC (2018) noted in their reporting that the EU chose to retaliate against orange juice, specifically mentioning that it “is a major export of Florida—a key US swing state.” Some of the states that bore the brunt of retaliatory tariffs included pivotal swing states like Michigan, New Hampshire, Ohio, and Wisconsin.

This political targeting did not go unnoticed, especially in the lead-up to the 2018 midterm elections. On September 18, 2018 President Trump tweeted that China was “attacking our farmers, ranchers, and industrial workers because of their loyalty to me...” In a speech to a think tank in October 2018, Vice President Pence also emphasized the link:

When it comes to influencing the midterms, you need only look at Beijing’s tariffs in response to ours... The tariffs imposed by China to date specifically targeted industries and states that would play an important role in the 2018 elections. By one estimate, more than 80 percent of US counties targeted by China voted for President Trump and I in 2016.^16^

In addition to messaging from Trump and Pence, the politically-targeted nature of the retaliation was also emphasized in the media. Typical of media coverage were headlines that made the link clear and explicit, such as “‘Chinese retaliatory tariffs aim to hit Trump in his electoral base” from the *Guardian* or “Beijing vows to retaliate in kind and has singled out products to maximize political pressure” from the *Los Angeles Times*.^17^

The prevalence of PTTR in the media is significant, since the media plays a critical role in influencing public attitudes toward foreign policy and shaping the information environment for voters.^18^ There are competing theories about how the media selects news coverage, ranging from them functioning as “a discrete strategic actor” shaping coverage to those who argue that the media primarily functions as a passive transmitter of elite views.^19^ When it comes to media coverage of international trade and investment, the media tends to report on events that negatively affect the home country.^20^ Wells et al. (2021, 224) find that the volume of media coverage of the trade war rose precipitously leading up to 2020 and that the tone became increasingly negative. Importantly for our analysis, the content emphasizing political targeting made up a significant portion of media coverage, and thus the information environment was replete with stories of PTTR.

While we have mentioned anecdotes of media coverage above, systematic analysis coverage shows that the political motivations behind retaliation received significant coverage. To assess the prevalence of stories reporting on the political targeting of tariff retaliation, we used Nexis Uni to search for newspaper stories and cable news coverage in the United States that discussed the trade war between January 1, 2018 to October 31, 2020. To ensure broad coverage of US media, we conducted two separate searches, with the search parameters detailed in Appendix A, which is available at the *Review of International Organizations’* webpage. The first search queried all US newspapers for articles that mentioned trade, retaliation, and tariffs. The search included some major national newspapers, but also included many local and regional newspapers, such as *The Citizens’ Voice* of Wilkes-Barre, Pennsylvania and *The Daily Iowan* from Iowa City, Iowa. Our search found more than 3,500 results, from which we coded a sub-sample. The second search focused on US newspapers and cable news transcripts with the broadest audience and yielded roughly similar results. By conducting the two searches, we insured that we had coverage of both local and national news sources. From our search results, we then coded whether the story specifically mentioned that certain states, politicians, or political parties were targeted, as detailed in Appendix A.

Table 1 shows examples from a variety of national news outlets, highlighting the directness and prominence with which outlets explained how foreign tariffs targeted specific regions. The language found in these excerpts highlights retaliation against both swing and base states: for instance, the *Wall Street Journal* coverage from March 21, 2018 emphasizes the targeting of Trump’s base, but its editorial coverage on June 25 also mentioned swing states. Also included in the table is a tweet from President Trump’s personal Twitter account, in which he also explicitly linked trade retaliation to political targeting of his base; the tweet itself also led to a wave of additional coverage of PTTR in outlets including *Bloomberg*, *Business Insider*, CNN, and NPR.

**Fig. 1:**
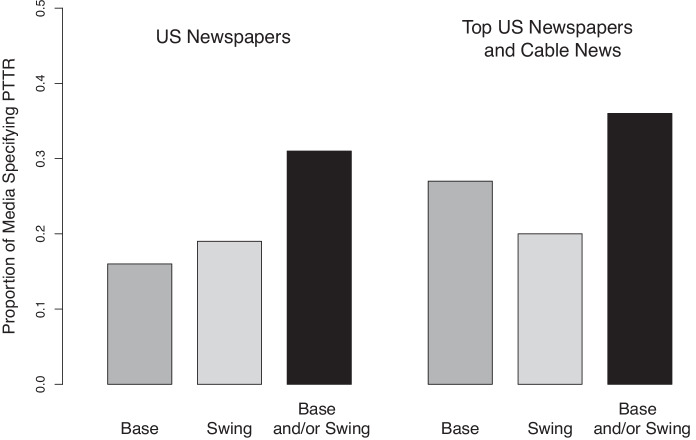
Media coverage specifying PTTR. The figure shows the the proportion of media sources that specifically mention that retaliatory tariffs targeted or specifically affected base or swing states. The base and swing state codings are not mutually exclusive, meaning an article may mention both. The left portion of the figure presents results for US newspapers searchable with Nexis Uni and the right portion presents results for a sub-sample of the largest US newspapers and cable news networks.

Results of the full media analysis are displayed in Figure 1. Of the coded sample from our first search, which looked at all US newspapers, 31 percent specifically mentioned base and/or swing state targeting. Our second search focused on US newspapers and cable news transcripts with the broadest audience and yielded roughly similar results.^21^ Of the sample coded from the second query, 36 percent specifically mentioned base and/or swing state targeting. Given that about a third of stories on the trade war in our sample specifically mentioned PTTR, the political aspects of the retaliation were well publicized to the mass public.

**Table 1:** Excerpts from media coverage

Date	Source	
March 2, 2018	*Politico*	...European Union officials are already planning retaliatory actions, targeting products from **politically sensitive Republican-run states**, including the imposition of tariffs on Harley-Davidsons made in Speaker Paul Ryan’s home state of **Wisconsin**; duties on bourbon made in Senate Majority Leader Mitch McConnell’s home state of **Kentucky**; and duties on orange juice from **Florida, a critical swing state**.
March 21, 2018	*Wall Street Journal*	China is preparing to hit back at trade offensives from Washington with tariffs aimed at President Donald Trump’s **support base**, including levies targeting US **agricultural exports from Farm Belt states**...
June 25, 2018	*Wall Street Journal* (editorial)	The damage is likely to have political consequences, as the retaliatory tariffs target **industries in swing states**. Wisconsin produces more than 90% of America’s ginseng, and 95% of that comes from Marathon County. The county went for Mr. Trump in 2016... Mr. Trump is also going to have some explaining to do to **Wisconsin cranberry farmers, Florida orange-juice producers, and Iowa soy and corn growers**...
November 9, 2018	*Washington Post*	China, Mexico and other foreign governments have retaliated by imposing their own tariffs on US exports, in many instances in areas where they might **hurt the president’s domestic political fortunes**. They targeted **Midwestern farms** that export to Chinese markets, Harley-Davidson motorcycles manufactured in the **Rust Belt**, and even bourbon produced in Senate Majority Leader Mitch McConnell’s home state of **Kentucky, among other businesses in Republican-controlled states.**
September 18, 2018	@realDonaldTrump	China has openly stated that they are trying actively trying to impact and change our election by attacking our **farmers, ranchers and industrial workers because of their loyalty to me**...

## Theory and existing research

Existing research most heavily emphasizes the theoretical relationship between trade and changing political behavior via economic voting.^22^ Voters engage in retrospective (or anticipatory) assessments of either their personal economic well-being or that of their community, state, neighborhood, or other relevant group (i.e., their sociotrope). They form a link between economic policies like the trade war and their economic welfare or the economic welfare of their sociotrope, then vote accordingly to reward or punish incumbent politicians. These models are an important part of our current understanding of the effects of targeted trade policies.

We depart from this common model of economic voting. Instead of looking at how trade policy affects the incumbent’s electoral fortunes via economic harm, we look at how politically-targeted trade retaliation can affect public opinion more broadly. Specifically, we theorize that PTTR can come to be viewed by the public as tool used by foreign powers to interfere in democratic elections. If our theory is correct, exposure to information about PTTR should increase worries about electoral interference.

Theoretical arguments about electoral interventions and interference can also help us understand reactions to PTTR, because more commonly-referenced types of electoral interference and partisan interventions share important features with PTTR. In introducing the extensive Partisan Electoral Intervention by the Great Powers dataset (PEIG), Dov Levin (2019) defines partisan electoral interventions as taking place when one country

... undertakes specific actions to influence an upcoming election in another sovereign country in an overt or covert manner which they believe will favor or hurt one of the sides contesting that election and which incurs, or may incur, significant costs to the intervener(s) or the intervened country (90).

PTTR fits the criteria underlying this definition. First, Levin describes electoral interfervention as encompassing acts that were “*intentionally* done in order to help or hurt one of the sides contesting the election for the executive” and “clearly carry significant costs.”^23^ PTTR carried out in response to the Trump trade war satisfies both of these criteria. Although China, the European Union, and other countries retaliating against the United States did not overtly declare that their targeting decisions were politically-motivated, this conclusion is supported both by systematic data and by anecdotal evidence. Fetzer and Schwarz (2020) and Kim and Margalit (2021) both examine the degree to which retaliation against Trump’s trade war was politically targeted and find that the tariffs were disproportionately levied at politically important states and regions. Fetzer and Schwarz (2020) find that trade retaliation measures from China, the European Union, Canada, and Mexico tended to be levied against goods produced in counties with higher levels of support for Republicans. Counties which “swung” for Trump (comparing his 2016 vote totals to the 2012 totals for Romney) were also more likely to be targeted by America’s trading partners.^24^ They note how the European Union uses an algorithm to choose tariff targets, one component of which likely includes the tariff’s effect on the foreign country’s political calculation. Kim and Margalit (2021) use a different measurement for exposure to retaliation and reach a similar conclusion. They find that a higher GOP vote share in the 2014 and 2016 House elections increased the degree to which Chinese tariffs targeted particular counties, and that this effect was even stronger in swing districts that were not starkly Democratic or Republican.

Qualitative interviews with representatives of associations and lobby groups in affected industries revealed that stakeholders believed they had been chosen for largely political reasons. For example, one interviewee affiliated with a large Midwestern soybean growers’ association described a clear perception among farmers that they had been targeted for political reasons: “I don’t think the question is ever really asked: ‘Why us? Why now?’ Farmers are astute. They understand the dynamics of the situation.” Another interviewee from a cranberry growing association said bluntly that “The growers see [retaliation] as political and [recognized] that the trade issues with China are much bigger than just cranberries.”^25^

Second, Levin also notes that an electoral intervention must entail “significant costs” to one or both parties. PTTR in the US-China trade war extracted a heavy economic and political toll. Fajgelbaum et al. (2020) show that the trade war resulted in a decline in real US wages and that this fell most heavily on Republican counties due to targeted retaliation. In addition to the economic impact, the political toll was especially high for Trump and the Republican Party. Blanchard et al. (2019) estimate that the Republican party lost five seats in the 2018 House elections due to retaliation against Trump’s trade war. Kim and Margalit (2021) find that counties with an additional 1% of workers exposed to Chinese retaliation saw declines of 0.26 percentage points for the Republican House candidate, compared to the preceding election. They estimate that retaliation from China cost Republicans four seats. They further supplement this aggregate analysis with survey research and analysis of Google search data, suggesting that voters “connected the dots” between Trump’s trade war and the anticipated economic harm of retaliation. Chyzh and Urbatsch (2021) and Wijesinghe (2020) also find a negative relationship between county-level soybean production and Republican vote shares in the 2018 midterm elections, which was caused by targeted retaliation.

During the Trump trade war, knowledge about PTTR was widely disseminated in the media. Senior Administration officials—most notably, President Trump and Vice President Pence—explicitly sought to link PTTR with rising concern about election interference in American elections. They did so by highlighting the same features of PTTR that also distinguish other forms of electoral interference, like its intended one-sided electoral effects.

Given this, we expect that public reactions to PTTR will be similar to those found in response to other forms of election interference. Trade policy that is designed with political motives, such as harming a specific political party or targeting electorally competitive areas, should increase public concern about foreign election interference in ways similar to those found in studies of other forms of election interference, such as side-taking by foreign powers, computer hacking, or ballot box tampering.

**Hypothesis 1.** ***Election Interference:*** *Learning that trade retaliation is politically targeted should increase concerns of electoral interference.*

### Partisan double standards hypotheses

How might respondents from different political parties perceive PTTR? Existing work on election interference, foreign endorsements, and sanctions suggests that respondents exhibit partisan double standards. They triangulate their reaction to foreign economic coercion or interventions based on their own personal preferences and partisan affiliations. If the foreign action is against a politician, party, or policy that the respondent supports, then they should react more negatively than if the foreign action targets an opposing political party or politician.

Tomz and Weeks (2020) posit three theoretical mechanisms that could explain partisan double standards. First, according to a “consequentialist” logic, interference is particularly galling to supporters of the targeted politician because they care most about the negative political consequences. If the interference causes their preferred candidate to lose, then they must suffer under the policies implemented by the opposing party. Supporters of the non-targeted politician are more tolerant of interference for the opposite reason: it helps their preferred candidate win. A second mechanism posits that supporters of the non-targeted politician underestimate the effect of interference that works in their favor, because of cognitive biases. The final mechanism is identity-based. Any interference targeting one’s preferred politician or in-group is viewed more negatively, irrespective of its actual impact on electoral competitions. Tomz and Weeks liken this to cheering for or against one’s favorite sports team. Citizens naturally get more angry when an outsider roots for their political opponent on the other “team.”

Tomz and Weeks (2020) and Bush and Prather (2020) both find evidence consistent with partisan double standards. The former conduct survey experiments that vary the presence and type of a foreign intervention against a hypothetical Presidential candidate in the United States. Americans reacted more negatively to electoral interference intended to harm their preferred candidate, compared to meddling against the other party. Democrats are substantially more likely to disapprove of election interference in the form of threats when the threat is carried out in support of a Republican (71% disapproving) than when it supports a Democrat (39%). Republicans showed a similar split: 71% disapproved of threats in support of a Democrat, while only 51% disapproved of threats which supported a Republican.^26^

Studying foreign side-taking endorsements, Bush and Prather (2020) find a partisan effect in both the United States and Tunisia. PTTR also fits their definition of side-taking, which “occurs when a country meddles in another country’s domestic politics in favor of a particular side” (3). When a foreign government endorses a respondent’s preferred candidate, the respondent is more supportive of economic engagement with the foreign country, compared with their level of support when the foreign country endorses the opposing side.

Corstange and Marinov (2012) find a similar effect using survey experiments in Lebanon. When a foreign country takes a partisan stance, supporting one side over another, this polarizes respondent attitudes regarding relations with the foreign country. They theorize that pre-existing partisan divides help amplify the polarizing effect of foreign side-taking, a background condition that is clearly present in the United States. Similar evidence has also been found in survey work in Russia and Ukraine that looks at Western economic sanctions and a trade dispute between Russia and Ukraine (Alexseev and Hale, 2020; Frye, 2019; Seitz and Zazzaro, 2019).^27^

The theoretical arguments for a partisan double standard in reactions to electoral interference should apply to both base and swing state PTTR. Applied to both forms of politically targeted trade retaliation, these arguments imply that members of the public will react in predictably partisan ways to PTTR. Those whose preferred party leaders are directly targeted should have the strongest negative reaction and express the greatest concerns about foreign interference.

In the context of President Trump’s trade war, learning that retaliatory tariffs were targeted with the goal of harming Trump’s political prospects—either through targeting his base or swing states—should generate the greatest concern about election interference among Republicans. By contrast, we would expect Democrats to have a more muted reaction, given that the retaliatory tariffs targeting the Republican base are politically advantageous for their preferred candidates.

**Hypothesis 2.** ***Partisan Effects:*** Supporters of the side targeted by PTTR (Base or Swing) should be most concerned about electoral interference.

## Research methods

To assess the effect of politically targeted trade retaliation on public attitudes, we employ a survey experiment that allows us to isolate the effects of different types of trade retaliation. We fielded our survey using Lucid Theorem on a diverse sample of over 3,500 respondents. The study was fielded between October 20th and 25th in 2020 and targeted respondents to resemble the demographics of US adults based on gender, age, geographic, and racial distributions. Survey samples from Lucid are increasingly used in social science research, including numerous articles published in top political science journals.^28^

While Lucid allows researchers to access a diverse sample of respondents, recent research finds that data quality declined as a result of the COVID-19 pandemic, so we employed attention checks and asked respondents to pledge to pay attention to address quality concerns. Approximately 36 percent of recruited participants failed the attention checks and were not included in the sample, which is generally consistent with broader trends for the time.^29^ Even though response quality declined during the pandemic, Peyton et al. (2020) find that studies conducted throughout the pandemic successfully replicated earlier studies and should generalize beyond the pandemic, though treatment effects from experiments fielded during the pandemic were somewhat more conservative given reduced attention of respondents. We also implemented the recommendation of Burleigh et al. (2018), and blocked respondents from participating if they were located outside of the US or were flagged for using a Virtual Private Server (VPS). The sample resulted in a diverse sample that closely mirrored the national population on demographics of age, partisanship, and gender, as shown in the demographic breakdown in Appendix B. Like most online surveys, our sample skews lower on income and is somewhat more likely to be college educated than the national population.

In designing the experiment, we chose a relatively hard test of our theory. The first critical decision was whether to focus the experimental vignette on trade retaliation by China, another country, or to leave the country unspecified. We wanted the scenario to be realistic as well as plausible, so we chose to use a real country that had retaliated against the US during the trade war.^30^ However, we also chose to make the test a hard test for the theory by focusing on the European Union, rather than China. At the time we fielded the survey, suspicions that China would engage in election interference were high. Both President Trump and Vice President Pence had accused China of attempting to interfere in US elections. Furthermore, they had also explicitly described China’s trade policy as an attempt at election interference. In addition to previously-mentioned remarks by Pence in October 2018, Trump also made the link explicit during a speech at the U.N. Security Council:

Regrettably, we found that China has been attempting to interfere in our upcoming 2018 election, coming up in November, against my Administration... They do not want me, or us, to win, because I am the first president ever to challenge China on trade, and we are winning on trade. We are winning at every level. We don’t want them to meddle or interfere in our upcoming election.

In order to make the test a hard one, we opted to focus on the trade policies of the European Union, a trading partner that had not been as strongly accused of using its trade policies to interfere in American elections. The test is also a harder one because most EU countries are allies of the United States who are not generally thought to interfere in US elections. Additionally, in the 2020 election, EU members had not publicly taken as strong of a stance against Trump, compared to the 2016 election. By contrast, China is viewed by many as a threat to the US, and it has engaged in cyber attacks and espionage against the US.^31^ Furthermore, public opinion toward China has become quite negative, with about two-thirds of Americans having an unfavorable view toward China,^32^ whereas a majority of Americans have a favorable view toward the EU.^33^ Thus, we would expect that respondents would be more sensitive to additional information about political targeting from a geostrategic adversary like China, compared to the EU, making ours a conservative test of the theory.^34^

Our survey experiment randomly assigned respondents to one of four conditions, each of which varied what the respondent read about the trade war. In the control condition, respondents read a few short lines about the trade war.

Control: In 2018 and 2019, the Trump Administration started a trade war by imposing tariffs on imports of steel and aluminum from the European Union.

These tariffs are meant to lower imports of steel from Europe into the United States and to convince the European Union to change its trade policies.

In response to President Trump’s tariffs, the European Union retaliated with tariffs against a variety of products that it imports from the United States.

The control treatment *does* specify that retaliation occurred, but *does not* specify any particular regional or political target of the retaliation. This allows us to differentiate between the public’s response to retaliation in general, versus retaliation that is politically targeted in the subsequent treatments.

Our study included three separate treatment conditions that described particular features of the retaliation. For respondents not assigned to control, they read the same information as in the control, and were randomly assigned to read one additional piece of information, which was either the Base, Swing, or Placebo treatment. We modeled the wording of these treatments after media reporting on the tariffs, so that our treatment in the survey experiment resembles the “treatment” received by respondents when they read information about the trade war or heard arguments from elites.

Base: ... The European Union intentionally chose products that come from states that voted for Trump in the last election. The European Union hopes that punishing these states will hurt President Trump politically and convince him to end the trade war.

Swing: ... The European Union intentionally chose products that come from “swing states” that will be important in the upcoming US election. The European Union hopes that punishing these states will hurt President Trump politically and convince him to end the trade war.

Placebo: ... The European Union announced the list of products in a press release and also communicated their decision to the United States Trade Representative through diplomatic channels. The official notification contained further details about the tariffs.

The Base and Swing conditions are the most important for our study. They each contain information about the politically-targeted nature of the European Union’s retaliation.^35^ The Base condition emphasizes how retaliation targeted states that supported President Trump in previous elections, and the Swing treatment emphasizes how retaliation targeted more electorally competitive areas in battleground states. Both treatments explain the intentionality behind the EU’s targeting, describing how the retaliation is meant to affect Trump’s decision making. When compared to the control condition, they allow us to isolate the effect of trade retaliation *being politically targeted* at the base or swing states, beyond the mere existence of retaliation itself.

As previously mentioned, we sought to design the treatments in a manner that made for a relatively hard test of the theory. One option would have been to model our treatments to mirror language used by President Trump or Vice President Pence that explicitly describes PTTR as a form of electoral interference. Rather than using such an explicit treatment, we chose more moderate language that only referenced the political nature of the trade retaliation (i.e., that the tariffs were targeted with political considerations in mind), but we avoided specifically linking the tariffs to electoral interference. The treatment thus presents respondents with facts about PTTR, but leaves it up to the respondent to decide on their own whether the fact that tariff policies were politically targeted should cause them to be worried about electoral interference.

The final treatment is a placebo which has a nearly identical word count to the Base and Swing treatments and a similar structure, but contains little information that would affect a respondent’s attitude about the trade war. We included this Placebo treatment to ensure that effects of the Base and Swing treatments resulted from the informational content of those treatments, as opposed to simply having additional information on the page. Like the Control treatment, the Placebo treatment describes the presence of retaliation but does not attribute any political motivations.^36^

We generally see balance across treatment groups in the characteristics of the respondents, as expected with random assignment. To formally assess balance, we used the approach described in Hansen and Bowers (2008) to compare each treatment with the control group. In each comparison, we fail to reject the null hypotheses of balance. We see some small differences between control and treatment in some covariates.^37^ For example, there are 5% more respondents identifying as Democrats in the Control group, compared to the Base treatment. Imbalance is very unlikely to influence our results. For starters, the estimates presented below do not change much when we include or exclude respondent characteristics.^38^ We also conducted sensitivity testing to show that imbalance in some other respondent characteristic would have to be many orders of magnitude larger than our currently observed levels of imbalance in order to affect our results.^39^

After reading about the trade war, respondents were presented a bullet-point recap of the key details of the treatment, which remained at the top of the survey screen as they answered post-treatment questions. For example, a respondent assigned to the Base treatment would have read:

To recap:

- The US put tariffs on imports from the EU to get them to change their trade policies.
- The EU retaliated with tariffs of their own on imports from the US.
- The EU’s retaliatory tariffs targeted President Trump’s base.

Our primary outcome measure comes from response to the following question:

I am worried the European Union’s retaliatory tariffs are an attempt to interfere with the upcoming US Presidential election.

Respondents could choose from five responses, ranging from “Strongly agree” to “Strongly disagree” with “Neither agree nor disagree” as a middle option. We deliberately chose the wording of this outcome measure. The term “election interference” has taken on a significant, negative connotation in the US context.^40^ This term, as used in media reports, has come to encompass things like leaking emails, hacking voting machines, or other malicious acts of election meddling. The outcome measure thus allows the respondent to independently make a connection between trade policy—an act that is not inherently electoral—and election interference, a much more serious and negative issue that directly impacts the well-being of democratic politics. If politically targeted trade policy makes respondents more likely to express worry about election interference, then that is more than just an answer that factually “connects the dots” from treatment to outcome. To agree with the prompt, respondents indicate more than just an acknowledgement that retaliation was politically targeted; they associate trade retaliation with the same term that is used to describe direct challenges to the foundations of democracy.

The wording of our dependent variable also raises the bar, since it requires that respondents express worry or anxiety about PTTR as a form of election interference. This is substantively important, since “political anxiety triggers engagement in politics” (Albertson and Gadarian, 2015, p. 1). Feeling worried or anxious about politics also leads people to seek out more information, especially when the information is threatening, such as PTTR (Gadarian and Albertson, 2014). Thus, if respondents are worried that PTTR is an attempt to interfere in the election, it implies that they not only believe the EU is attempting to interfere, but that the respondent is likely to find it politically salient, since the information induces a sense of anxiety or worry.

## Results

We progress through our analysis in two parts. First, we analyze whether our respondents are worried about PTTR as a form of election interference. Specifically, we test whether the Base and Swing treatments result in respondents being worried that the EU’s retaliatory tariffs are an attempt to interfere with the election. We then analyze the moderation hypotheses, testing whether there is a partisan double standard in the public’s concern over PTTR as a form of election interference.

### Hypothesis 1: Effect of treatment on fears of interference

**Fig. 2:**
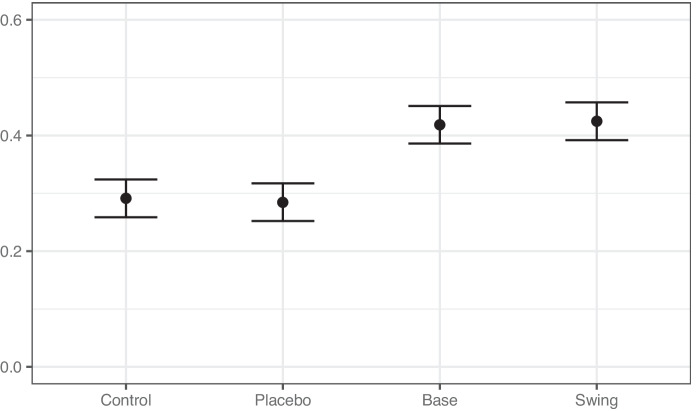
Politically targeted trade retaliation perceived as election interference. The figure shows the proportion of respondents in each condition who are somewhat or strongly worried the European Union’s retaliatory tariffs are an attempt to interfere with the upcoming US Presidential election. Lines show 95 percent confidence intervals.

The main effects of our treatments are displayed in Fig. 2, which shows the proportion of respondents who are worried that the EU’s tariff retaliation is an attempt to interfere in the 2020 presidential election. The left side of the figure shows that 29 percent of the respondents in the control condition are worried that the retaliatory tariffs are an attempt at election interference. This shows that even generic trade retaliation in the lead-up to the 2020 election was viewed by some as a concerning form of election interference. We also find that the placebo condition does not change the public’s concern ($p = 0.78$), which gives us confidence that the treatment effects reported in the other conditions are not driven by the length of text of the treatments.

When comparing the Base and Swing treatments to the control, we find strong support for our first hypothesis. Each of the treatments specifying politically targeted tariff retaliation result in substantively large and significant effects for the full sample, as shown on the right side of Figure 2. The Base and Swing treatments each lead to a 13 percentage point increase in the number of respondents who express concern about election interference ($$p<0.01)$$. These results clearly demonstrate that politically targeted trade retaliation is viewed as a distinct form of foreign interference than generic trade retaliation and a substantial portion of the public is concerned that PTTR is attempted election interference.

These results were robust to a variety of other specifications, in addition to our baseline comparisons. In the Appendix, section. C.1 replicates all analyses controlling for respondents’ characteristics (e.g. age, income, etc). In section C.2, we also tested whether respondents reacted differently to information about the trade war *without* specifying that the other country retaliated. We also replicate results excluding respondents who said they neither agreed nor disagreed with the statement about election interference, shown in Online C.3. The main results are robust to these specification changes.

### Hypothesis 2: Partisan double standards

Hypothesis 2 predicts that reactions to different types of PTTR should vary by respondent party affiliation. Existing research would predict that Republicans should react more negatively to both the Base and Swing treatments than Democrats.

**Fig. 3:**
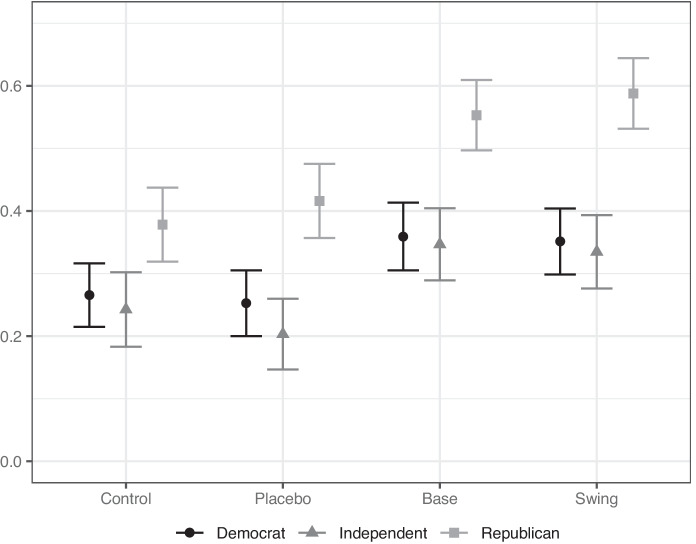
Politically targeted trade retaliation perceived as election interference, by party. The figure shows the proportion of respondents in each condition who are somewhat or strongly worried the European Union’s retaliatory tariffs are an attempt to interfere with the upcoming US Presidential election. Respondents are separated based on whether they self-identified as a Democrat, Republican, or something else (categorized as Independent). Lines show 95 percent confidence intervals.

**Fig. 4:**
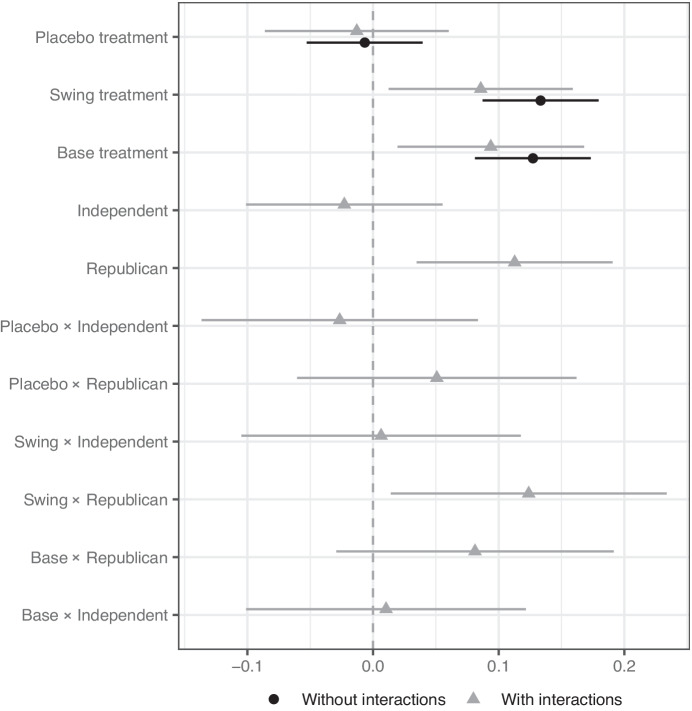
Partisan interactions on worries about election interference. The figure shows the effect of our treatments on whether a respondent is worried the European Union’s retaliatory tariffs are an attempt to interfere with the upcoming US Presidential election. Respondents are separated based on whether they self-identified as a Democrat, Republican, or something else (categorized as Independent). Lines show 95 percent confidence intervals.

We show our results broken down by party in two ways. Figure 3 shows the proportion of respondents worried about electoral interference by treatment condition and party. Figure 4 shows results from a linear regression of a binary variable measuring whether respondents are worried about the retaliation being election interference or not on indicators for the various treatment conditions and their interactions with indicator variables for identification as a Republican or Independent. Democrats are the base category. The figure shows the change in the predicted probability that a respondent is worried about election interference compared to the baseline control for Democrats. The figure reports the treatment effects for models with and without interactions between the treatment and partisanship identification.

In Figure 3 we show that Republicans (grey squares) have a higher baseline concern about trade retaliation being a form of election interference than either Democrats or Independents, in all treatment conditions. It is noteworthy that both forms of PTTR increase concern about election interference for *both* parties. Even among Democrats, both types of PTTR increase worries about election interference. In existing work on partisan double standards, the partisan gap usually arises because the beneficiary of the foreign intervention reacts little, while the target of the intervention reacts very negatively. Here, we find that even the ostensible beneficiaries of PTTR—Democrats—associate base and swing targeting with the negative concept of electoral interference.

However, Figure 4 shows that the magnitude of treatment effects did *not* always vary substantially by party. Interestingly, we do not find support for the first part of Hypothesis 2—that the Base treatment will trigger differing reactions across parties. Republicans and Democrats alike reacted negatively to tariffs targeting states that Trump previously won. The third line of Figure 4 shows how the Base treatment increased worry of election interference among Democrats. And looking at the second line from the bottom, Republicans have a slightly larger reaction to the Base treatment, compared to Democrats or Independents, but we cannot reject the null hypothesis that the treatment effect is the same for Republicans and Democrats. Even when retaliation targeted states that Trump won in 2016, Democrats had heightened concerns about election interference and the Republican reaction was not significantly larger. Political targeting of the Republicans’ base generates heightened concern about election interference across the political spectrum, not just among Republicans.

Turning to the Swing treatment, it too increased worry about election interference for both Republicans and Democrats, and the difference in the effects across party are consistent with Hypothesis 2. The positive and significant interaction effect for Republicans (third line from the bottom) shows that the negative reaction to the Swing treatment was even larger for Republicans than for Democrats. In substantive terms, we find that the Swing treatment increases the number of Republicans expressing concern by 21 percentage points (*p* < 0.01), whereas it only increases concern among Democrats by 6 percentage points (*p *= 0.05).

Why would we observe a larger partisan double standard for the Swing treatment than the Base treatment? The answer could lie in the different mechanisms for partisan reactions. Swing state targeting could be especially prone to trigger a partisan double standard according to a consequentialist logic, where respondents care most about the political fortunes of their preferred candidate. The political consequences of targeting partisan strongholds—base targeting—are likely to be minimal in systems like the United States’, since a large percentage of voters would have to vote across party lines to change the electoral outcomes. Targeting the incumbent’s base might activate feelings based on identity; Republicans don’t like any action against their co-partisans. However, targeting the base is not likely to change the outcome of elections. On the other hand, targeting swing states may shift electoral outcomes even if only a small percentage of voters in those states are influenced. Like base targeting, swing state targeting activates feelings based on identity and antipathy towards anyone targeting your group or your preferred politician. However, swing state targeting has the added component of placing one’s preferred candidate’s electoral chances at risk. Since we fielded our survey near the 2020 election, consequentialist concerns could have been more at the front of respondents’ minds. This proximity would also likely make Democratic disapproval of PTTR weaker, which makes it even more surprising to see Democratic respondents reacting negatively to both treatments.

Further research would be needed to disentangle these different mechanisms, but our results suggest that consequentialist arguments are a stronger explanation for partisan reactions than those based only on a shared identity with those being targeted. Given the timing of our survey near a major election, we would have thought that partisan double standards were likely. Since the Base treatment *still* fails to elicit a partisan double standard, this suggests that mechanisms based solely on shared identity with the targeted groups do not necessarily lead to partisan interpretations of outside interference.

We also investigated whether there were partisan double standards applied to a different outcome measure—a feeling thermometer towards the retaliating party. In general, the results were similar with those shown here. Partisan splits were biggest under the Swing treatment, with Republicans subsequently expressing the greatest increase in dislike towards the EU.^41^

We also examined whether treatment effects differed by the respondents’ locations. Theoretically, a respondent living in a swing or base state might react more negatively to treatments highlighting how foreign countries targeted them with retaliation. In the online appendix, we show that there is some, though generally weak, evidence that treatment effects differed across states. For example, Republicans living in base states reacted more negatively to the Base treatment. However, sufficient power for detecting these heterogeneous treatment effects would require a much larger sample size than ours.^42^

## Conclusion

As the public becomes increasingly aware of the specter of foreign interference in democratic elections, the possibility grows that foreign trade policies and other tools of economic statecraft will come to be viewed as a form of foreign interference. American politicians such as Donald Trump and Michael Pence have explicitly linked politically-targeted trade retaliation with election interference.

We contribute two important findings to the understanding of politically-targeted trade retaliation and its links with electoral interference. First, our results underscore that the costs of economic coercion should not be limited to the economic damage or short-term partisan victories. Our evidence clearly shows that a significant portion of Americans are worried that politically-targeted trade retaliation constitutes a form of electoral interference. A complete accounting of the costs of the United States’ recent trade war must include this impact on public opinion, and the damage that politically-targeted trade retaliation has done to public trust in democratic institutions.

Second, even during a time of heightened party polarization, both Democrats and Republicans reacted strongly and negatively towards PTTR. Members of both parties expressed significant worries about PTTR, despite the fact that they were told that PTTR was designed to hurt President Trump politically. Although we find suggestive evidence that Republicans reacted slightly more negatively, our findings make clear that Americans’ suspicion of PTTR is not a purely partisan issue. Instead, we find that Americans across the political spectrum view PTTR as a concerning form of foreign intervention.

These findings suggest promising avenues for future research. Recent evidence suggests that PTTR “works”: targeted political pressure succeeds in hurting the political fortunes of the targeted political party (Kim and Margalit, 2021; Blanchard et al., 2019). However, if PTTR is increasingly viewed as electoral interference, this runs the risk that sending states will find themselves the victim of negative backlash. This raises several intriguing questions for future study. First, if popular backlash serves to limit the feasibility of PTTR strategies, the applicability of PTTR may differ by regime type. While democracies with clearly deliniated geographic voting constituencies may be seen as easier targets for PTTR, the risks of provoking a popular backlash could limit the perceived efficacy of PTTR against democracies. Future research could explore how regime type affects both vulnerability towards PTTR as well as the propensity to engage in it versus other states.

Second, these findings could suggest an optimistic possibility in which fears of provoking backlash will limit the future use of PTTR. A more concerning alternative is that PTTR remains a commonly-used tool of international politics and further contributes to declining trust in democratic institutions. Determining which is more likely will require more research to better understand whether and how states take into account potential public backlash in considering whether or not to engage in PTTR.

## Supplementary Information

Below is the link to the electronic supplementary material.

Supplementary file1 (PDF 482 kb)

Supplementary file2 (R 41 kb)

Supplementary file3 (CSV 418 kb)

Supplementary file4 (XLSX 17 kb)

## Data Availability

The dataset generated by the survey research analyzed for the current study are available in the Dataverse repository and the *Review of International Organizations*’ webpage.

## Footnotes

1 Chyzh and Urbatsch (2021)

2 Fajgelbaum et al. (2020); Blanchard et al. (2019)

3 Zheng, Sarah. April 5, 2018. “How China hit Donald Trump’s supporters where it hurts as tariffs target Republican Party’s heartlands.” *South China Morning Post*. https://www.scmp.com/news/china/diplomacy-defence/article/2140464/how-china-hit-donald-trumps-supporters-where-it-hurts

4 Barro, Josh. September 11, 2019. “Trump’s proposed wine and cheese tariffs may be his most normal trade policy,” *New York Magazine*, https://nymag.com/intelligencer/2019/09/trumps-cheese-tariffs-may-be-his-most-normal-trade-policy.html; Swanson, Anna. September 3, 2019. “How Trump just made your dinner more expensive,” *New York Times*. https://www.nytimes.com/2019/10/03/business/trump-tariffs.html

5 One former US trade official described the products selected as part of a “yuppie list”. Author interview, 2021; see also Johnson, Oswald. January 30, 1987. “Pact half stiffs US tariffs on European luxury goods,” *Los Angeles Times*, https://www.latimes.com/archives/la-xpm-1987-01-30-mn-1748-story.html

6 Wagner, Marcus and Weihuan Zhou. August 20, 2021. “It’s hard to tell why China is targeting Australian wine. There are two possibilities,” *The Conversation*. https://theconversation.com/its-hard-to-tell-why-china-is-targeting-australian-wine-there-are-two-possibilities-144734

7 Blanchard et al. (2019); Chyzh and Urbatsch (2021); Fajgelbaum et al. (2020); Kim and Margalit (2021).

8 European Commission Press Release, 23 March 2021. “Strengthening the EU’s autonomy – Commission seeks input on a new anti-coercion instrument.” https://ec.europa.eu/commission/presscorner/detail/en/IP_21_1325. Accessed 7 December 2021.

9 Tomz and Weeks (2020); Bush and Prather (2020).

10 Lee, Carol E. and Courtney Kube. October 4, 2018. “Pence presses White House case on China’s election ‘meddling’,” *NBC News*. https://www.nbcnews.com/politics/national-security/pence-presses-white-house-case-china-election-meddling-n916696

11 Ibid.

12 Tomz and Weeks (2020); Bush and Prather (2020)

13 Congressional Research Services. 2019. “Retaliatory Tariffs and US Agriculture.” https://fas.org/sgp/crs/misc/R45903.pdf.

14 While Georgia ended up switching from Trump to Biden in 2020, it was not generally viewed as a likely swing state in 2017.

15 Report cited in Marcellus (2020).

16 Lee and Kube, *NBC News*

17 The Guardian (2018); Los Angeles Times (2018)

18 Baum and Potter (2008); Druckman (2005)

19 Baum and Potter (2008) 40; Bennett (1990)

20 Brutger and Strezhnev (2018); Guisinger (2017).

21 The specific sources are detailed in Appendix A.

22 e.g., Jensen et al. (2017); Mansfield and Mutz (2009)

23 Emphasis in original. Note that the PEIG data focus on interference from the United States and Russia, so actions against the United States during Trump’s trade war are not included.

24 While Trump likely had his own political reasons for wanting to amplify perceived antagonism from abroad and divert attention from his other political troubles, the data suggest he was not entirely wrong.

25 Authors’ interviews, summer 2020.

26 We used their results for the “threats” condition since it most closely fits with PTTR. Threats “combine an endorsement with a promise of future reward or threat of future punishment, such as threatening to downgrade future relations if the preferred candidate loses” (859). While PTTR is not made explicitly contingent upon the outcome of a future policy, it does seek to inflict political pain in response to a given policy, and thus mirrors a threat which has been carried out. Similar results obtain for other operations.

27 Two notable exceptions find mixed effects of sanction targeting on respondent reactions. One of the few exceptions to the polarization hypothesis, Sejersen (2021) finds that partisan targeting can lower disapproval of sanctions. Using survey experimental data from Venezuela, he finds that targeting sanctions at the incumbent regime blunts backlash, as citizens perceive sanctions which specifically target regime supporters entailing less collateral damage against everyday Venezuelans. Conversely, Shulman and Bloom (2012) argue that in Ukraine, partisan targeting raised overall disapproval of the sanctions, since it implied an even greater intrusion on national elections and identity.

28 For examples of political science articles using Lucid, see Tomz and Weeks (2020), Kim and Margalit (2021), and Margalit and Solodoch (2019).

29 Aronow et al. (2020).

30 Using highly salient real actors actors can invoke stronger treatment effects in some situations, though Brutger et al. (2020) find that many experimental results replicate across a broad range of actors.

31 Zeng and Li (2019).

32 https://www.pewresearch.org/global/2020/04/21/u-s-views-of-china-increasingly-negative-amid-coronavirus-outbreak/ This is also consistent with the public viewing China as a trade adversary (Sattler and Schweinberger, 2019).

33 https://www.pewresearch.org/fact-tank/2019/10/21/attitudes-toward-eu-are-largely-positive-both-within-europe-and-outside-it/

34 Survey research also tends to find higher support for protectionism against Asian countries compared to European ones. For example, Di Tella and Rodrik (2020) found large differences in support for tariffs depending on whether an adverse economic shock came from France versus Cambodia.

35 Across both treatments, we avoided mentioning specific states or industries targeted by retaliation, since that might induce confounding if respondents inferred additional information beyond our desired treatments (Dafoe et al., 2018). Additionally, none of the treatments involve deception; the content of the Base and Swing treatments was present in media discourse and in the thinking of real-world stakeholders in affected industries.

36 We are also aware that experimentalists have to consider the potential for demand effects, however they “are usually a potential problem only when they are positively correlated with the true experimental objectives’ predictions” (Zizzo , 2010), which is unlikely to be the case in this experiment. Additionally, any such expectations would have also appeared in the control and placebo conditions, potentially altering the baseline, but the treatment effects should not be altered since there is not an obvious social desirability related to responses.

37 It is also to be expected that some differences can arise when comparing 16 covariates across 3 treatment groups, for a total of 48 comparisons.

38 See Appendix C.

39 Chaudoin et al. (2018); Cinelli et al. (2020). For the full description of the balance tests and sensitivity testing, see Appendix B.

40 Our choice to ask respondents about worry regarding Europe’s attempt to “interfere” with the election reflects the more common use of “election interference” in the media and the public, as opposed to worry about Europe engaging in an election “intervention — a term often used in scholarly research. A search of Google Trends for “election AND interference” and “election AND intervention” from the start of 2018 up until November 2020, shows interference search generates more than double the average interest than the intervention search. Additionally, to the extent that we are interested in capturing worries, “interference” is a higher bar, requiring respondents to link PTTR to the term with a more negative association, which makes it a higher bar to find a significant effect in our study.

41 See Appendix D.

42 See Appendix, section C.5 for results and power calculations.

## References

[CR1] Albertson, B., & Gadarian, S. K. (2015). *Anxious politics: Democratic citizenship in a threatening world*. Cambridge University Press.

[CR2] Alexseev MA, Hale HE (2020). Crimea come what may: Do economic sanctions backfire politically?. Journal of Peace Research.

[CR3] Aronow, P. M., Kalla, J., Orr, L., & Ternovski, J. (2020). Evidence of rising rates of inattentiveness on lucid in 2020. *SocArXiv*

[CR4] Baum MA, Potter PB (2008). The relationships between mass media, public opinion, and foreign policy: Toward a theoretical synthesis. Annu. Rev. Polit. Sci..

[CR5] BBC. (2018). *US tariffs: Allies retaliate with levies on jam, lamps and sleeping bags*. Technical Report BBC.

[CR6] Bennett WL (1990). Toward a theory of press-state. Journal of communication.

[CR7] Blanchard, E. J., Bown, C. P., & Chor, D. (2019). *Did Trump’s Trade War Impact the 2018 Election?*. Technical Report National Bureau of Economic Research.

[CR8] Brutger, R., Kertzer, J. D., Renshon, J., Tingley, D., & Weiss, C. M. (Forthcoming). Abstraction and detail in experimental design. *American Journal of Political Science*

[CR9] Brutger, R., & Strezhnev, A. (Forthcoming). International disputes, media coverage, and backlash against international law. *Journal of Conflict Resolution*

[CR10] Burleigh, T., Kennedy, R., & Clifford, S. (2018). How to screen out VPS and international respondents using qualtrics: A protocol. *Available at SSRN 3265459*

[CR11] Bush SS, Prather L (2020). Foreign meddling and mass attitudes toward international economic engagement. International Organization.

[CR12] Chaudoin S, Hays J, Hicks R (2018). Do we really know the wto cures cancer?. British Journal of Political Science.

[CR13] Chyzh, O. V., & Urbatsch, R. (2021). Bean counters: The effect of soy tariffs on change in republican vote share between the 2016 and 2018 elections. *The Journal of Politics*, *83*.

[CR14] Cinelli, C., Ferwerda, J., & Hazlett, C. (2020). sensemakr: Sensitivity analysis tools for OLS in R and Stata. *Available at SSRN 3588978*.

[CR15] Corstange D, Marinov N (2012). Taking sides in other people’s elections: The polarizing effect of foreign intervention. American Journal of Political Science.

[CR16] Dafoe A, Zhang B, Caughey D (2018). Information equivalence in survey experiments. Political Analysis.

[CR17] Di Tella R, Rodrik D (2020). Labour market shocks and the demand for trade protection: Evidence from online surveys. The Economic Journal.

[CR18] Druckman JN (2005). Media matter: How newspapers and television news cover campaigns and influence voters. Political communication.

[CR19] Fajgelbaum PD, Goldberg PK, Kennedy PJ, Khandelwal AK (2020). The return to protectionism. The Quarterly Journal of Economics.

[CR20] Fetzer, T., & Schwarz, C. (2020). Trade wars and politics: Evidence from Trump’s trade wars. *The Economic Journal*.

[CR21] Frye T (2019). Economic sanctions and public opinion: Survey experiments from russia. Comparative Political Studies.

[CR22] Gadarian SK, Albertson B (2014). Anxiety, immigration, and the search for information. Political Psychology.

[CR23] Guisinger, A. (2017). *American opinion on trade: Preferences without politics*. Oxford University Press.

[CR24] Hansen, B. B., & Bowers, J. (2008). Covariate balance in simple, stratified and clustered comparative studies. *Statistical Science,* 219–236.

[CR25] Jensen, J. B., Quinn, D. P., & Weymouth, S. (2017). Winners and losers in international trade: The effects on US Presidential voting. *International Organization*, *71*, 423–457.

[CR26] Kim SE, Margalit Y (2021). Tariffs as electoral weapons: The political geography of the us-china trade war. International organization.

[CR27] Levin DH (2019). Partisan electoral interventions by the great powers: Introducing the peig dataset. Conflict Management and Peace Science.

[CR28] Los Angeles Times. (2018). *Chinese retaliatory tariffs aim to hit Trump in his electoral base*. Los Angeles Times.

[CR29] Mansfield, E. D., & Mutz, D. C. (2009). Support for free trade: self-interest, sociotropic politics, and out-group anxiety. *International Organization*, *63*, 425–457. Publisher: Cambridge University Press.

[CR30] Marcellus, S. (2020). *Key swing states are among hardest hit by China’s tariffs*. Technical Report Yahoo Finance.

[CR31] Margalit, Y., & Solodoch, O. (2021) Against the flow: Differentiating between public opposition to the immigration stock and flow. *British Journal of Political Science,* 1-21.

[CR32] Peyton, K., Huber, G. A., & Coppock, A. (2020). The generalizability of online experiments conducted during the covid-19 pandemic. https://osf.io/preprints/socarxiv/s45yg/. Accessed 7 May 2022.

[CR33] Sattler, T., & Schweinberger, T. (2019). Trade as a foreign policy issue: A bilateral micro perspective. *Unpublished manuscript, last modified October*, *22*.

[CR34] Seitz, W., & Zazzaro, A. (2019). Sanctions and public opinion: The case of the Russia-Ukraine gas disputes. *Review of International Organizations,* 1–27.

[CR35] Sejersen M (2021). Winning hearts and minds with economic sanctions? evidence from a survey experiment in venezuela. Foreign Policy Analysis.

[CR36] Shulman, S., & Bloom, S. (2012). The legitimacy of foreign intervention in elections: the Ukrainian response. *Review of International Studies,* 445–471.

[CR37] The Guardian. (2018). *Chinese retaliatory tariffs aim to hit Trump in his electoral base*. Technical Report The Guardian.

[CR38] Tomz M, Weeks JL (2020). Public opinion and foreign electoral intervention. American Political Science Review.

[CR39] Wells, R., Zeng, K., & Wilkins, A. (2021). Media coverage of chinese investment in the United States: Politics and missed opportunities. *Newspaper Research Journal* (p. 07395329211013962).

[CR40] Wijesinghe, A. S. (2020). Retaliatory tariff and 2018 mid term election: Was there an effect of Chinese soybeans tariff?

[CR41] Zeng K, Li X (2019). Geopolitics, nationalism, and foreign direct investment: Perceptions of the china threat and american public attitudes toward chinese fdi. The Chinese Journal of International Politics.

[CR42] Zizzo DJ (2010). Experimenter demand effects in economic experiments. Experimental Economics.

