# Supplementary material for: Trade Wars and Election Interference
Source: Rev Int Organ. 2022 Jun 11;18(1):1–25. doi: 10.1007/s11558-022-09464-2 (PMC9187851; doi:10.1007/s11558-022-09464-2)
Supplement: Supplementary file 1 — Supplementary file1 (PDF 482 kb) [file 11558_2022_9464_MOESM1_ESM.pdf]

Appendix Sections

Appendix A: Media Coverage of PTTR

Appendix B: Sample Method and Demographics

B.1 Summary Statistics for Sample

B.2 Balance Across Treatment Groups

B.3 Sensitivity Testing

Appendix C: Additional Analyses

C.1 Results including respondent characteristics

C.2 Analysis using No Retaliation as Control

C.3 Analysis excluding “Neither agree/disagree”

C.5 Residency interactions and power analysis

Appendix D: Opinions about the EU

A Appendix: Media Coverage of PTTR

To assess the prevalence of media coverage of PTTR, we used Nexis Uni to search for newspaper articles in the United States from January 1, 2018 to October 31, 2020. To limit the universe of results to those related to the tariff escalation and relation associated with the trade war, we used the search terms "trade AND retal* AND tariff*". This first search, when restricted to the United States and Newspapers, yielded over 3,500 results. Nexis Uni sorts results by relevance, but we also manually checked that each article was about the trade war. We then selected the first 100 results for coding.[43] This sample resulted in a broad range of stories across the date range specified and represented a diverse set of sources including local, regional, and national newspapers.

The second search used the same search terms and date range, but restricted the sample to newspapers with the highest readership and also cable news transcripts. To determine high readership newspapers, we used the 2013 Alliance for Audited Media's 2013 Snapshot Report. From the report we selected the top-25 paid newspapers with the highest average circulation (both print and digital). We then searched those that were available through Nexis Uni, which resulted in the following sources:

- Wall Street Journal Abstracts
- The New York Times
- USA Today
- Los Angeles Times
- Daily News (New York)
- The New York Post
- The Washington Post
- The Orange County Register
- Tampa Bay Times
- The Philadelphia Inquirer
- Star Tribune (Minneapolis)

[43] We removed duplicate appearances that resulted from the source issuing a correction to the initial story.

- The Atlanta Journal-Constitution
- The Mercury News (San Jose)

Additionally, we also searched cable news transcripts from ABC, CBS, and NBC.

Articles were coded as “Base” if they:

- Mention base states affected/targeted by name (e.g. "producers in Mississippi will be hit hard"). These states were Alabama, Alaska, Arkansas, Idaho, Indiana, Kansas, Kentucky, Louisiana, Mississippi, Missouri, Montana, Nebraska, North Dakota, Oklahoma, South Carolina, South Dakota, Tennessee, West Virginia, and Wyoming.[44]

- If article mentions specific politician being targeted, code the party of the politician and then code the state they represent [Base, Swing, or nothing if neither], or if the article refers to the politician representing the republican base, stronghold, etc

- Those that mention affecting/targeting “Republicans”, "base", Trump’s supporters, areas that carried Trump, etc.

Articles were coded as “Swing” if they:

- Mention swing states affected/targeted by name. These states were Arizona, Colorado, Florida, Georgia, Maine, Michigan, Minnesota, Nevada, New Hampshire, North Carolina, Pennsylvania, Virginia, and Wisconsin.[45]

- Mentions "swing", "competitive" districts, states that flipped, etc that are affected/targeted

- If article mentions specific politician being targeted, code the party of the politician and then code the state they represent [Base, Swing, or nothing if neither], or if the article refers to the politician representing a “swing state", “contested state", etc.

---

[44] States were coded as “Base” if former President Trump won with greater than 10 percent margin in 2016.

[45] States were coded as “Swing” if former President Trump won with less than a 6 percent margin in 2016. Both swing and base codings are similar to those in existing work. Kim and Margalit (2021) use a 10% cutoff criteria (eg vote share for one party is 0.4 - 0.6) for categorizing swing areas. Blanchard, Bown and Chor (2019) use competitiveness bins, based on presidential vote share (<0.3, 0.3-0.4, 0.4-0.5, 0.5-0.6, 0.6-0.7, >0.7). Around the 50% vote share, this is equivalent to a 10% cutoff criteria. Ma and McLaren (2018) define swing states using a 5% and a 10% cutoff criteria.

Additional Article Excerpts

In the main manuscript, we chose a subset of excerpts. To give additional context, we include another set of illustrative examples here, in Table A.1.

Table A.1: Additional examples of politically-targeted trade retaliation

Date	Source	
March 9, 2018	*The Atlantic*	The [European retaliation] choices are not random, especially given upcoming midterm elections in the US. . . Matthew Oxenford, a researcher on transatlantic economic relations at the London-based Chatham House, told me. "That's very consciously being done with **an eye towards influencing a particular political player**. Florida oranges is another thing because **Florida is always a close state in US elections**.
May 31, 2018	*Washington Post*	Stung by the US action, the allies quickly hit back. The EU said it would impose tariffs on **politically sensitive items like bourbon from Senate Majority Leader Mitch McConnell's home state of Kentucky.**
June 21, 2018	*New York Times*	The European Union fought back. . . against the Trump administration's tariffs, slapping penalties on an array of American products that **target the president's political base**, like bourbon, motorcycles, and orange juice.
June 22, 2018	*BBC*	Many of the products the EU has in its sights are specifically **chosen to have maximum political effect.** Bourbon whiskey is produced in **Kentucky, the state of Senate Majority Leader Mitch McConnell.**
June 24, 2018	*The Guardian*	Both China and the EU have been politically motivated in their choice of US products to target. Take the **swing-state of Wisconsin**. . . Harley-Davidsons are manufactured in the state. Or take the largest item on the Chinese list due to take effect in two weeks—soybeans. They are grown in **Iowa and Nebraska, both Trump states**; bourbon on the EU list comes from **Kentucky, another Trump state**, home of the Senate majority leader, Mitch McConnell. Chinese tariff hikes on oranges will hit growers in the **swing-state of Florida**.
September 3, 2018	*New York Times*	Beijing hopes it can convince these voters—and their elected representatives—that the president's trade war could hurt them. Over the summer, the Chinese took aim at Mitch McConell, the Senate majority leader. In his home state, **Kentucky**, 18,000 jobs depend on whiskey. So they put a 25 percent tariff on it. Representative Paul D. Ryan, the House speaker, is from **Wisconsin**, a leading producer of cranberries. So cranberries were added to the list, for good measure. And China went after pork and soybeans, two of the leading farm products in **Iowa, home of [Republican] Charles E. Grassley, a powerful member of the Senate Agricultural Committee**.

B Appendix: Sample Demographics

B.1 Summary Statistics for Sample

Table B.1 shows summary statistics for various respondent characteristics for our sample, in the first column. The second column shows those same characteristics from the 2019 US Census data. For partisan identification, we used data from Pew, which covers registered voters for 2018/2019. Our sample tracked relatively closely with the broader US population on most measures. Our sample included slightly fewer older respondents, and the average income of our sample was below US averages. Both are common features of online surveys like ours. Our respondents had somewhat higher levels of education.

Demographic	Portion of Sample	US Population
Age 18 to 24	0.128	0.132
Age 25 to 39	0.278	0.266
Age 40 to 59	0.312	0.325
Age >60	0.251	0.293
Female	0.520	0.510
Household income $0 to $50,000	0.510	0.371
Household income $50,001 to $100,000	0.297	0.288
Household income $100,001 to $150,000	0.105	0.156
Household income >$150,000	0.088	0.185
Republican	0.312	0.290
Democrat	0.374	0.330
Independent (includes non-partisan or other)	0.314	0.380
Four-year college degree	0.372	0.317

Table B.1: Study demographics. US population information on age, sex, income, and education are from the Census Bureau and are for 2019. Partisan identification is from Pew and covers registered voters for 2018/9.

B.2 Balance Across Treatment Groups

Here, we compare balance across treatment groups in respondent characteristics. We do this using a series of pairwise comparisons, between respondents in the control group and then each of the three treatment groups. Table B.2, Table B.3, and Table B.4 shows the means of each variable,

for the control group and the swing, base, and placebo treatment groups respectively. Below each table, we report the omnibus χ^2 test statistic and p-value from the approach developed in Hansen and Bowers (2008).

For each pairwise comparison, we fail to reject the null, implying overall balance in respondent characteristics, between control and treatment. The approach developed in Hansen and Bowers (2008) is generally more conservative than an approach based on a logistic regression of treatment on covariates. In that approach, the researcher regresses treatment (logistic) on a constant and all covariates, then on a constant alone. The researcher then uses a likelihood ratio test to assess whether they can reject the null hypothesis that the reduced model (without covariates) fits the data better. When using that approach, we also fail to reject the null, which implies balance. The p-values associated with those χ^2 test statistics are: 0.22, 0.16, and 0.18.

The results from the Hansen and Bowers (2008) and logistic regression approach are very similar since our sample size contains approximately 875 respondents per treatment group. The comparisons between control and treatment are thus based on approximately $N = 1,750$ which is over 100 times the number of covariates being assessed for balance.

Two individual comparisons across treatment and control yield significant p-values at 0.05 level. There are 5% less people in one of the middle income brackets for the control group, compared to those in the base treatment. There are also 5% more Democrats in the control group compared to the base group. Our finding of only two statistically significant differences at the $p < 0.05$ level is roughly what one would expect when making $16 \times 3 = 48$ pairwise comparisons. These two differences are also substantively small and highly unlikely to influence results. In the case of difference in proportion of Democrats, we conduct subset analysis by party for treatment effects, in the main manuscript, which conditions on that variable.

Table B.2: Balance test between control group and swing treatment

	Control Prop.	Swing Prop.	Diff.	SD of Diff.	Z-score	
Age 18 to 24	0.16	0.15	-0.01	0.02	-0.38	
Age 25 to 39	0.27	0.29	0.01	0.02	0.59	
Age 40 to 59	0.31	0.33	0.01	0.02	0.60	
Age >60	0.26	0.25	-0.01	0.02	-0.48	
Female	0.51	0.51	-0.01	0.03	-0.23	
Male	0.49	0.49	0.01	0.03	0.23	
Household income $0 to $50,000	0.53	0.50	-0.03	0.03	-1.09	
Household income $50,001 to $100,000	0.27	0.31	0.04	0.02	1.96	.
Household income $100,001 to $150,000	0.11	0.11	0.00	0.02	0.25	
Household income >$150,000	0.10	0.08	-0.02	0.01	-1.49	
Republican	0.30	0.33	0.03	0.02	1.15	
Democrat	0.41	0.37	-0.03	0.02	-1.42	
Independent (includes non-partisan or other)	0.29	0.30	0.01	0.02	0.35	
College graduate	0.38	0.38	-0.00	0.02	-0.02	
Swing state resident	0.36	0.31	-0.05	0.02	-1.91	.
Base state resident	0.19	0.18	-0.01	0.02	-0.49	

Overall test $\chi^2 = 15.6$ with 13 degrees of freedom ($p = 0.269$). Significance codes: $\cdot = 0.1$, $* = 0.05$, $** = 0.01$.

Table B.3: Balance test between control group and base treatment

	Control Prop.	Base Prop.	Diff.	SD of Diff.	Z-score	
Age 18 to 24	0.16	0.13	-0.03	0.02	-1.73	.
Age 25 to 39	0.27	0.31	0.04	0.02	1.60	
Age 40 to 59	0.31	0.31	-0.00	0.02	-0.01	
Age >60	0.26	0.24	-0.01	0.02	-0.62	
Female	0.51	0.52	0.00	0.03	0.12	
Male	0.49	0.48	-0.00	0.03	-0.12	
Household income $0 to $50,000	0.53	0.48	-0.05	0.03	-1.84	.
Household income $50,001 to $100,000	0.27	0.32	0.05	0.02	2.31	*
Household income $100,001 to $150,000	0.11	0.10	-0.00	0.02	-0.31	
Household income >$150,000	0.10	0.10	-0.00	0.01	-0.14	
Republican	0.30	0.33	0.03	0.02	1.29	
Democrat	0.41	0.35	-0.05	0.02	-2.10	*
Independent (includes non-partisan or other)	0.29	0.31	0.02	0.02	0.92	
College graduate	0.38	0.36	-0.02	0.02	-0.66	
Swing state resident	0.36	0.33	-0.03	0.02	-1.13	
Base state resident	0.19	0.19	0.00	0.02	0.10	

Overall test $\chi^2 = 19.2$ with 13 degrees of freedom ($p = 0.116$). Significance codes: $\cdot = 0.1$, $* = 0.05$, $** = 0.01$.

Table B.4: Balance test between control group and placebo treatment

	Control Prop.	Placebo Prop.	Diff.	SD of Diff.	Z-score	
Age 18 to 24	0.16	0.13	-0.03	0.02	-1.72	.
Age 25 to 39	0.27	0.30	0.03	0.02	1.13	
Age 40 to 59	0.31	0.34	0.02	0.02	0.92	
Age >60	0.26	0.25	-0.01	0.02	-0.32	
Female	0.51	0.52	0.01	0.03	0.40	
Male	0.49	0.48	-0.01	0.03	-0.40	
Household income $0 to $50,000	0.53	0.49	-0.03	0.03	-1.36	
Household income $50,001 to $100,000	0.27	0.31	0.04	0.02	1.79	.
Household income $100,001 to $150,000	0.11	0.11	0.00	0.02	0.03	
Household income >$150,000	0.10	0.09	-0.01	0.01	-0.48	
Republican	0.30	0.30	-0.00	0.02	-0.15	
Democrat	0.41	0.38	-0.03	0.02	-1.15	
Independent (includes non-partisan or other)	0.29	0.32	0.03	0.02	1.37	
College graduate	0.38	0.38	0.00	0.02	0.14	
Swing state resident	0.36	0.33	-0.03	0.02	-1.17	
Base state resident	0.19	0.17	-0.02	0.02	-1.25	

Overall test $\chi^2 = 17.3$ with 13 degrees of freedom ($p = 0.185$). Significance codes: $\cdot = 0.1$, $* = 0.05$, $** = 0.01$.

B.3 Sensitivity Testing

The presence of imbalance in characteristics that we did measure/observe raises the possibility of imbalance across treatment/control groups in some unobserved characteristic, which could affect our estimates, in theory. Sensitivity testing is a powerful tool for assessing the severity of these types of threats. Sensitivity approaches vary, but they generally ask some form of the following question: How bad would imbalance in unobservable characteristics across treatment groups need to be, compared to imbalance in observed characteristics, in order to substantially change estimated treatment effects? For an International Relations-specific application, see Chaudoin, Hays and Hicks (2018).

Cinelli, Ferwerda and Hazlett (2020) provide a useful tool for visualizing this thought exercise. Unobservables have greater potential to influence treatment effect estimates as they become more correlated with treatment and the outcome of interest (as in standard intuition about omitted variable bias). Figure B.1 shows contour plots for these potential relationships, based on partial R^2 statistics. The horizontal axis shows the strength of a possible relationship between an unobservable and treatment; the vertical axis shows the strength of the possible relationship between the unobservable and the outcome. Each contour shows a possible treatment effect estimate, in the hypothetical world in which an unobservable had particular relationships with treatment and outcome. The triangle in the bottom left shows our estimated treatment effect, of the Base treatment (OLS coefficient $= 0.12$). The red dashed line shows the contour where our estimated treatment effect is driven to zero by the presence of unobservables. However, to reach this contour, an unobservable would need to be much more imbalanced across treatment and control *and* have much more explanatory power than our observables. To see this, the red dot shows the location of our most imbalanced observable (Democrat) in this two-dimensional space. An unobservable would have to be much more imbalanced and powerful in order to drive our estimates to zero. And recall that the variable Democrat was our most imbalanced observable.

Figure B.2 shows the same thought exercise for our estimated Swing treatment effects. For the comparison between the Swing treatment and the Control group, income was our most imbalanced

observable. Here, too, unobservables would have to be much more imbalanced and powerful than anything in our sample in order to drive estimated treatment effects to zero.

Figure B.1: Sensitivity Contour Plot, Base Treatment

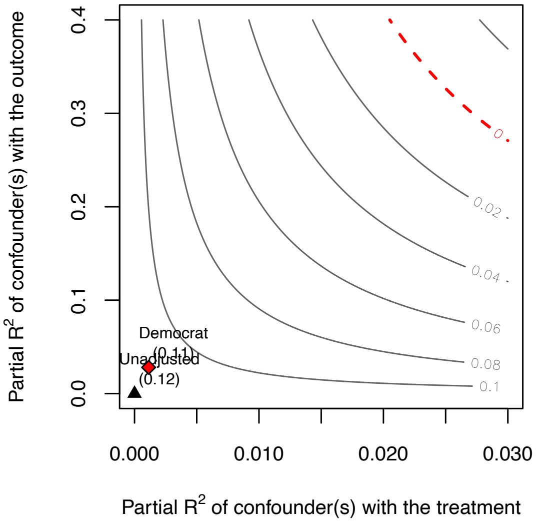

Figure B.2: Sensitivity Contour Plot, Swing Treatment

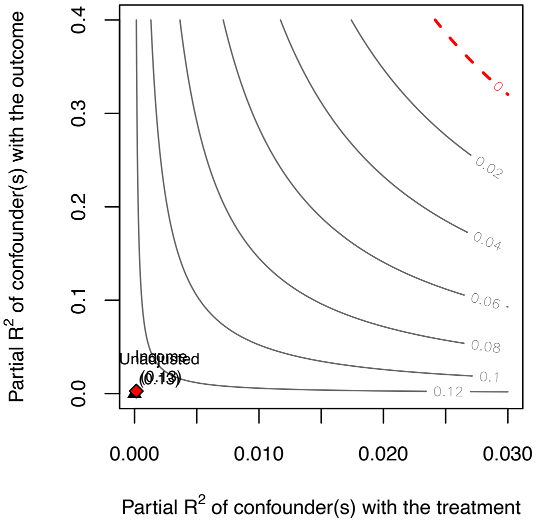

C Appendix: Additional Analyses

C.1 Results including respondent characteristics

Table C.1 shows results from regressing the electoral interference outcome variable on treatment indicators and a set of respondent characteristics. Column 1 is the analogue of the regression used to generate Figure 2 in the main manuscript. Column 2 is the analogue of the regression for Figure 3. Columns 3 and 4 are the analogues of **??**, which uses the EU thermometer measure as the outcome variable.

Results are very similar to those presented in the main table, which is to be expected given random treatment assignment. For worry about election interference, both the Base and Swing treatments have positive and significant effects, with the Placebo treatment not having a significant effect. The Swing treatment is strongest for Republicans. For feelings towards the EU, the only significant treatment effect is for the interaction with the swing treatment and Republican.

C.2 Analysis Using "No Retaliation" Condition

The main manuscript compared outcomes between the control condition where the EU engaged in tariff retaliation (without it being politically targeted) with various forms of PTTR in the treatment conditions. We also included an additional treatment condition, which did not include any references to the existence of EU retaliation at all, which read as follows:

> In 2018 and 2019, the Trump Administration started a trade war by imposing tariffs on imports of steel and aluminum from the European Union.
>
> These tariffs are meant to lower imports of steel from Europe into the United States and to convince the European Union to change its trade policies.

This treatment was included because it played a role in analyzing outcomes not focused on in this paper. Recall that the outcome question asked respondents to react to "I am worried the European Union's retaliatory tariffs are an attempt to interfere with the upcoming US Presidential election." This meant that the variable of interest, which specifically mentioned retaliatory tariffs,

Table C.1: Effect of treatment, with controls

	Worries of Election Interference		Feeling Towards EU	
	(1)	(2)	(3)	(4)
Placebo	−0.005	−0.011	1.157	0.685
	(0.023)	(0.037)	(1.180)	(1.885)
Swing	0.129***	0.088**	−0.668	0.195
	(0.023)	(0.037)	(1.180)	(1.894)
Base	0.119***	0.086**	−1.678	−1.248
	(0.023)	(0.038)	(1.182)	(1.920)
Independent	−0.015	−0.017	−12.147***	−14.249***
	(0.020)	(0.040)	(1.033)	(2.037)
Republican	0.196***	0.132***	−18.178***	−15.492***
	(0.021)	(0.040)	(1.056)	(2.039)
Male	0.041**	0.040**	0.650	0.765
	(0.017)	(0.017)	(0.855)	(0.855)
Employed	0.043**	0.042**	2.397**	2.471***
	(0.018)	(0.018)	(0.935)	(0.936)
White	−0.060**	−0.059**	−0.653	−0.738
	(0.024)	(0.024)	(1.228)	(1.228)
Black	0.038	0.039	−2.796*	−2.878*
	(0.032)	(0.032)	(1.640)	(1.641)
Income	0.013***	0.013***	0.635***	0.649***
	(0.005)	(0.005)	(0.231)	(0.231)
Age	−0.001***	−0.001***	−0.124***	−0.122***
	(0.001)	(0.001)	(0.027)	(0.027)
College graduate	−0.016	−0.016	4.076***	4.025***
	(0.019)	(0.019)	(0.974)	(0.974)
Placebo x Independent		−0.024		2.513
		(0.056)		(2.841)
Placebo x Republican		0.049		−0.908
		(0.056)		(2.865)
Swing x Independent		0.016		3.502
		(0.057)		(2.878)
Swing x Republican		0.117**		−5.986**
		(0.056)		(2.830)
Base x Independent		0.020		2.296
		(0.057)		(2.875)
Base x Republican		0.087		−3.590
		(0.056)		(2.847)
Constant	0.263***	0.285***	65.490***	65.143***
	(0.037)	(0.040)	(1.865)	(2.053)
Observations	3,171	3,171	3,154	3,154
R^2	0.073	0.074	0.127	0.130
Note:				*p<0.1; **p<0.05; ***p<0.01

was not well-aligned with the no-retaliation treatment, so it is omitted from the main analysis of our study.

To be thorough, though, we replicate the above analyses using this alternative condition without retaliation as the baseline, rather than the retaliation condition. The advantage of using the No Retaliation treatment as the baseline, is that we can evaluate whether non-targeted trade retaliation (the Control condition) generates higher concerns about election interference than the No Retaliation treatment. As shown in Figure C.1, there is not a significant difference in worries about election interference between the Control and No Retaliation conditions, which gives us greater confidence that the main results are driven by PTTR, and not retaliation in general.

Figure C.1: Politically Targeted Trade Retaliation Perceived as Election Interference, with No Retaliation Treatment

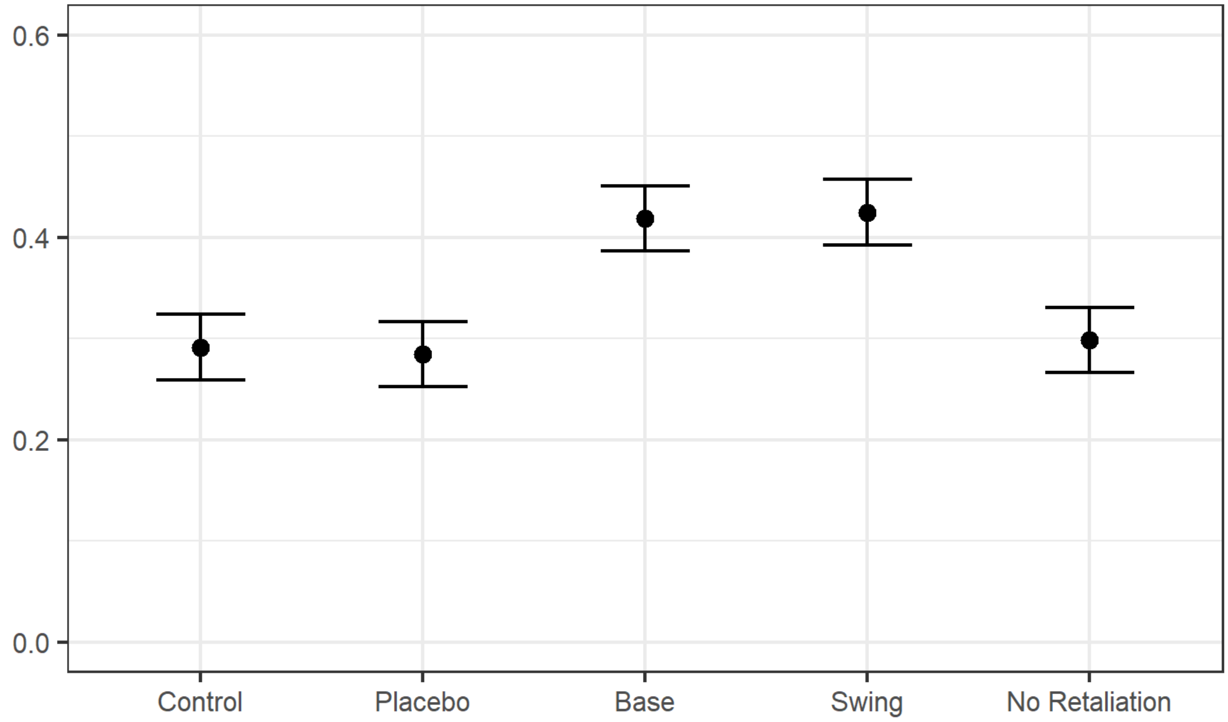

The figure shows the proportion of respondents in each condition who are somewhat or strongly worried the European Union's retaliatory tariffs are an attempt to interfere with the upcoming US Presidential election. Lines show 95 percent confidence intervals. This figure includes the No Retaliation treatment.

C.3 Analysis Excluding "Neither agree/disagree"

Table C.2 replicates the main analysis, only it excludes respondents who answered "neither agree nor disagree" for the question about whether they worried about election interference. Results are again similar to the main analysis, with no sign or significance changes pertaining to the main arguments above.

Table C.2: Effect of treatment on worries of election interference, dropping neither agree/disagree

	Worried about election interference	
	(1)	(2)
Placebo treatment	0.012	−0.022
	(0.029)	(0.045)
Swing treatment	0.205***	0.132***
	(0.029)	(0.046)
Base treatment	0.176***	0.127***
	(0.029)	(0.046)
Independent		−0.017
		(0.049)
Republican		0.147***
		(0.048)
Placebo × Independent		0.029
		(0.071)
Placebo × Republican		0.080
		(0.069)
Swing × Independent		0.069
		(0.072)
Swing × Republican		0.129*
		(0.068)
Base × Independent		0.056
		(0.071)
Base × Republican		0.075
		(0.068)
Constant	0.413***	0.371***
	(0.021)	(0.032)
Observations	2,256	2,227
R^2	0.034	0.075
Note:	*p<0.1; **p<0.05; ***p<0.01	

C.4 Residency interactions and power analysis

Figure C.2 shows the results where we interact each of the treatments with whether the respondent lived in a swing state or base state. We split the sample by party identification and then plotted the effects of each treatment, swing/base, and their interactions, by party. We coded swing states as those whose 2016 election margin was within 6% (Arizona, Colorado, Florida, Georgia, Maine, Michigan, Minnesota, Nevada, New Hampshire, North Carolina, Pennsylvania, Virginia, and Wisconsin). We coded base states as those that Trump won by 10% or more in 2016 (Alabama, Alaska, Arkansas, Idaho, Indiana, Kansas, Kentucky, Louisiana, Mississippi, Missouri, Montana, Nebraska, North Dakota, Oklahoma, South Carolina, South Dakota, Tennessee, West Virginia, and Wyoming). In general, we do not find differences across party, treatment, and state. Republicans living in base states reacted with less worry about election interference to the Base treatment, compared to Republicans living in states that were neither base nor swing states, i.e. states that voted more Democratic.

Table C.3: Number of observations across treatments, residency state, and partisanship

		Treatment				
Residency	Party	Control	Base	Swing	Placebo	Total
Base	Democrat	51	47	45	30	173
Base	Independent	46	39	41	55	181
Base	Republican	58	71	63	50	242
Swing	Democrat	110	88	89	110	397
Swing	Independent	85	89	80	78	332
Swing	Republican	90	89	86	78	343
Other	Democrat	163	149	165	161	638
Other	Independent	104	123	122	128	477
Other	Republican	90	104	116	110	420
**Total**		797	799	807	800	3203

While the triple interaction among residency state, partisanship, and treatment is theoretically of interest, splitting our sample in this way results in $3 \times 3 \times 4 = 36$ individual cells. Any resulting

Figure C.2: Effect of residency state on worries of election interference

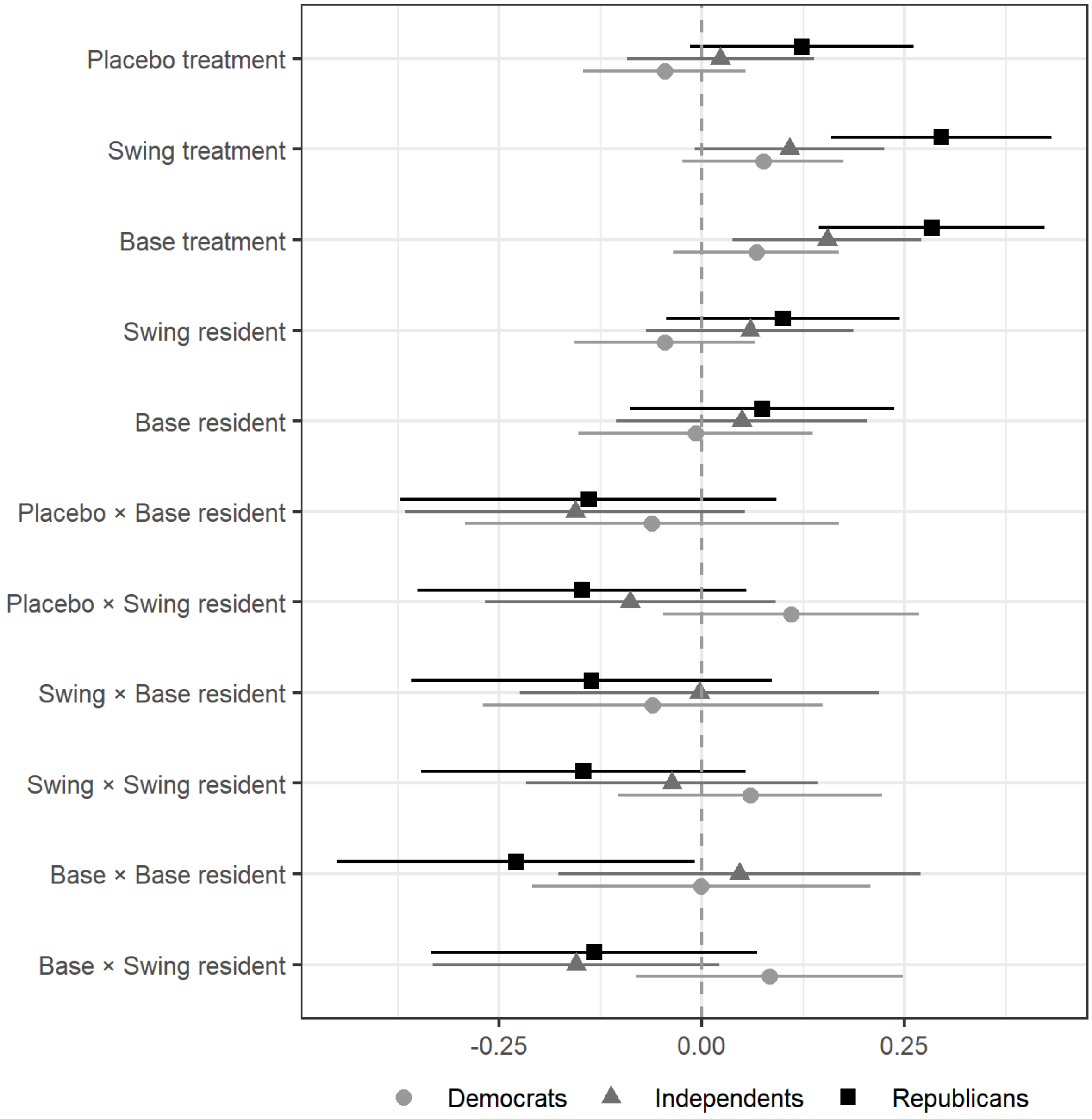

The figure shows the interaction of state of residency with treatment on worries of election interference. There is a statistically significant and negative effect for Republicans living in base states exposed to the base treatment.

analysis is likely significantly under-powered, and so we do not speculate about the effects of such interactions. Note that the sample size in Table C.3 does not equal that in the main analysis as some respondents declined to respond to the question which asked for their partisan identification.

To conduct power analysis, we used the approach described in Blair et al. (2019). We supposed that we had an even simpler survey design than that used here. We considered a design with only two treatments, a control group and a swing treatment. We then supposed that we wanted to detect an interaction effect between living in a swing state and the swing state treatment. We assumed that one third of respondents lived in a swing state.

We then calculated the sample size necessary to detect an interaction term between living in a swing state and the swing state treatment (successfully reject the null of equivalent effects, with at least 80% probability). Each colored line in Figure C.3 corresponds to a different "true" size for the interaction effect. The plot then shows the power to detect that particular interaction term effect as the sample size changes. If the interaction term were -0.5, then we would need a sample size of at least 500 to achieve 80% power.

What effect size is reasonable? To benchmark, we used our estimated interaction term effect size for Republicans $\times$ Swing treatment, from Figure 4. There, we estimated that the interaction term between Republican and Swing treatment was 0.13. This is a generous (non-conservative) way to benchmark our power analyses, because this was one of the largest and the only statistically significant interaction term effect we found for the partisan analyses.

If the "true" effect size for the swing treatment and swing state residency interaction term were similar, then we would need a very large sample to achieve 80% power. The green and orange lines at the bottom of Figure C.3 show how weakly power increases with sample size at that treatment effect size.

Figure C.3: Power analysis

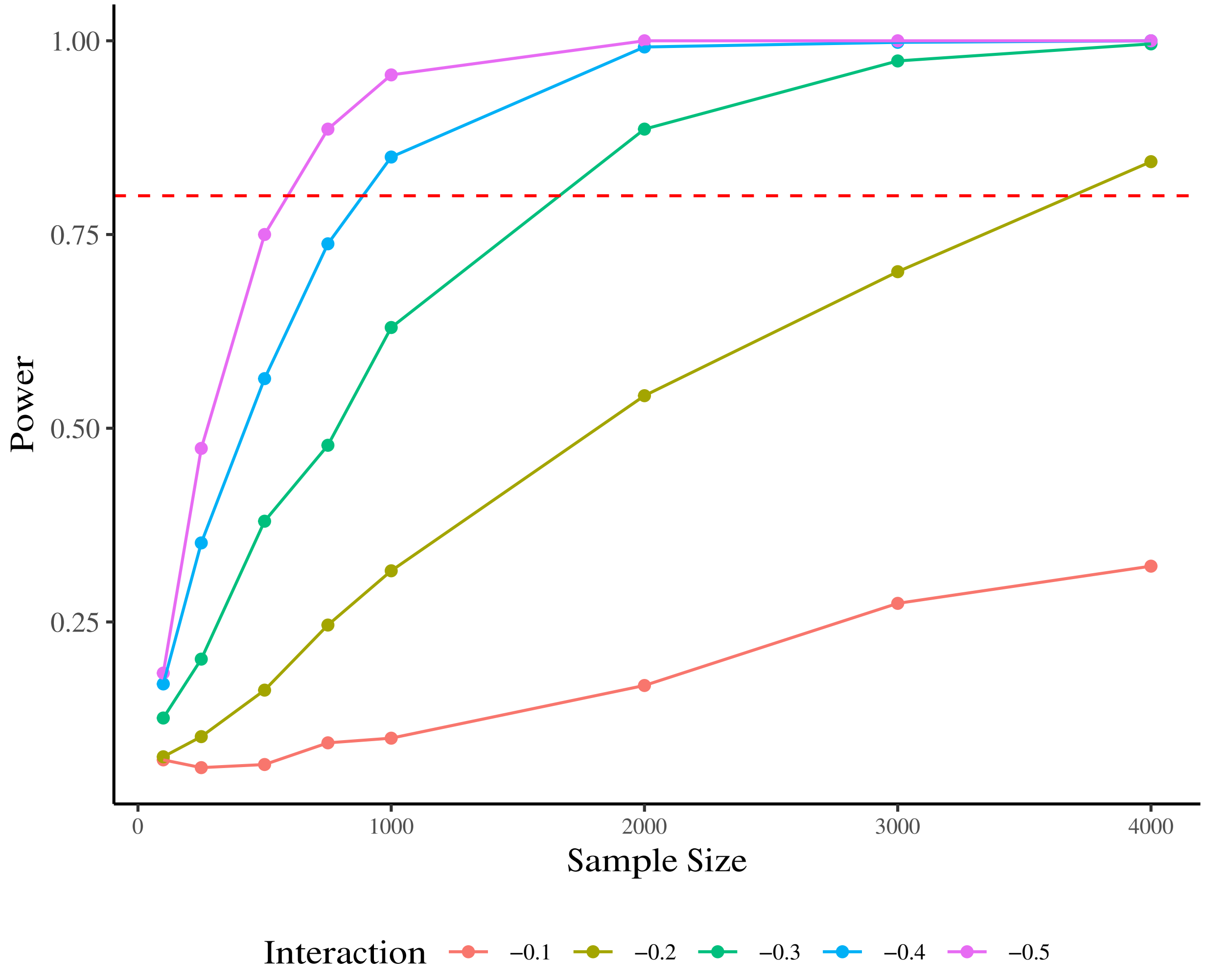

The lines correspond to different sized interaction term effects, for the interaction between an indicator for swing state residents and a swing state treatment indicator.

C.5 Potential age cohort effects

As we note in the main article, the European Union's imposition of PTTR during the 2018–2020 period was not the first time they have done so. Specifically, during the 2003–4 period, some European countries enacted retaliatory tariffs in response to the George W. Bush Administration's imposition of steel tariffs. Because of this, it is possible that Americans who were old enough to remember this earlier round of tariffs may react differently (more negatively) to the imposition of European retaliation during the Trump administration. To explore this, we compared the reactions between those who were adults in 2003 with those were younger. Those who were over 35 in 2020 would have been over 18 in 2023.

We do not find that there is a significant interaction ($p < 0.05$) between our treatments and respondents being over 35 at the time of our study.

Figure C.4: Potential heterogeneity by age cohort

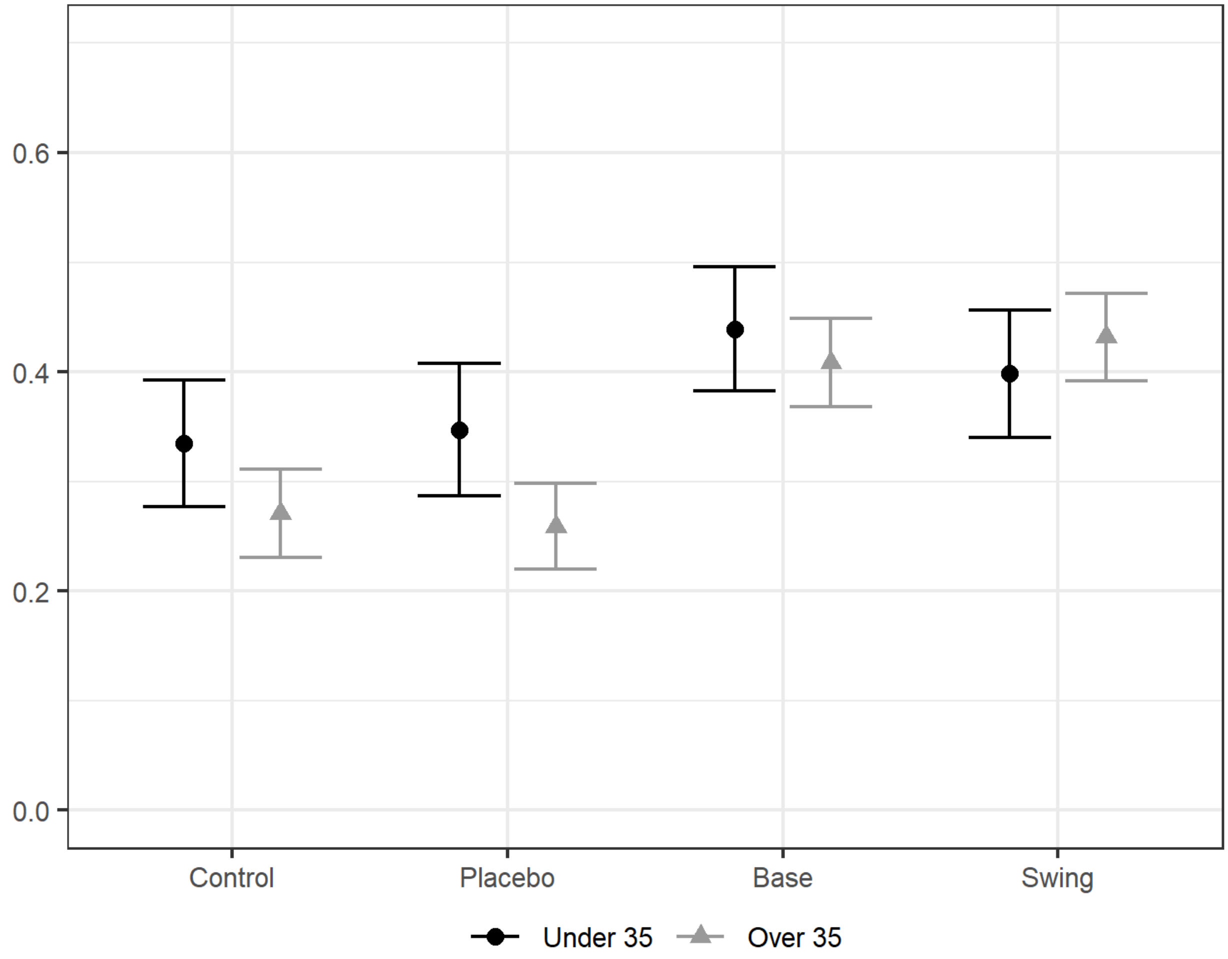

The figure shows the interaction of age with treatment on worries of election interference. We do not find any significant differences between those who were adults during the 2003–4 US–EU trade dispute (i.e., over than 35 in 2020). This figure does not include control variables.

Table C.4: Effect of treatment, with age cohort

	Worries of Election Interference	
	(1)	(2)
Placebo	0.013	0.007
	(0.043)	(0.041)
Swing	0.064	0.068*
	(0.042)	(0.041)
Base	0.104**	0.102**
	(0.041)	(0.040)
Over 35	−0.063*	−0.015
	(0.036)	(0.042)
Placebo × Over 35	−0.025	−0.015
	(0.051)	(0.050)
Swing × Over 35	0.097*	0.091*
	(0.051)	(0.050)
Base × Over 35	0.033	0.026
	(0.051)	(0.049)
Constant	0.335***	0.285***
	(0.030)	(0.043)
Controls?	No	Yes
N	3,176	3,171
R^2	0.022	0.074

*p < .1; **p < .05; ***p < .01

The second model includes controls for partisanship, gender, employment status, race, income, age, and education.

D Appendix: Opinions about the EU

We also analyzed respondents' feelings about the EU. We expected that learning that trade retaliation is politically targeted will lead to less favorable opinions of the EU, and that Republicans would respond more negatively according to the logic of partisan double standards. The results are generally consistent with those highlighted in the main manuscript, regarding worries about election interference. We replicated the same regressions used in the main manuscript, only the outcome measure is a 0-100 point feeling thermometer about how the respondent feels towards the EU.

?? shows results from regressing the feeling thermometer measure on the treatment indicators, with and without partisan interaction terms. In the first column, only the Base treatment has a significant, negative effect on feelings towards the EU. The second column shows that there were only partisan double standards with the Swing treatment. Among Republicans, the effect of the Swing treatment was significantly larger than among Democrats.

Table D.1: Attitude towards EU (0 to 100 feeling thermometer)

	0–100 Feeling Thermometer	
	(1)	(2)
Placebo treatment	1.061	0.410
	(1.240)	(1.902)
Swing treatment	−1.194	0.025
	(1.241)	(1.907)
Base treatment	−2.160*	−1.143
	(1.240)	(1.935)
Independent		−14.968***
		(2.036)
Republican		−15.582***
		(2.029)
Placebo x Independent		3.218
		(2.862)
Placebo x Republican		−0.516
		(2.891)
Swing x Independent		3.345
		(2.896)
Swing x Republican		−5.211*
		(2.851)
Base x Independent		2.460
		(2.899)
Base x Republican		−3.433
		(2.874)
Constant	54.896***	64.096***
	(0.879)	(1.321)
Observations	3,232	3,182
R^2	0.002	0.101
Note:	*p<0.1; **p<0.05; ***p<0.01	
